# Strategic Trastuzumab Mediated Crosslinking Driving Concomitant HER2 and HER3 Endocytosis and Degradation in Breast Cancer

**DOI:** 10.7150/jca.32470

**Published:** 2020-03-05

**Authors:** Jennifer Mary Wymant, Edward John Sayers, Duncan Muir, Arwyn Tomos Jones

**Affiliations:** 1 School of Pharmacy and Pharmaceutical Sciences, Cardiff University, Redwood Building, King Edward VII Avenue, Cardiff, CF10 3NB; 2 School of Earth and Ocean Sciences, Cardiff University, Main Building, Park Place, Cardiff, CF10 3AT

**Keywords:** HER2, crosslinking, endocytosis

## Abstract

Efficacious anticancer therapies for targeting plasma membrane receptors with antibody based therapeutics are often contingent on sufficient endocytic delivery of receptor and conjugate to lysosomes. This results in downregulation of receptor activity and, in the case of antibody-drug conjugates (ADCs), intracellular release of a drug payload. The oncogenic receptor HER2 is a priority therapeutic target in breast cancer. Known as an “endocytosis resistant” receptor, HER2 thwarts the receptor downregulating efficiency of the frontline treatment trastuzumab and reduces the potential of trastuzumab-based therapies such as trastuzumab-emtansine. We previously demonstrated that strategically inducing trastuzumab and HER2 crosslinking in breast cancer cells promoted endocytosis and lysosomal delivery of the HER2-trastuzumab complex, stimulating downregulation of the receptor. Here we reveal that HER3, but not EGFR, is also concomitantly downregulated with HER2 after crosslinking. This is accompanied by strong activation of MEK/ERK pathway that we show does not directly contribute to HER2/trastuzumab endocytosis. We show that crosslinking induced trastuzumab endocytosis occurs via clathrin-dependent and independent pathways and is an actin-dependent process. Detailed ultrastructural studies of the plasma membrane highlight crosslinking-specific remodelling of microvilli and induction of extensive ruffling. Investigations in a cell model of acquired trastuzumab resistance demonstrate, for the first time, that they are refractory to crosslinking induced HER2 endocytosis and downregulation. This implicates further arrest of HER2 internalisation in developing trastuzumab resistance. Overall our findings highlight the potential of receptor crosslinking as a therapeutic strategy for cancer while exposing the ability of cancer cells to develop resistance via endocytic mechanisms.

## Introduction

Human epidermal growth factor receptor 2 (HER2) belongs to the ErbB family of receptor tyrosine kinases and is a critical therapeutic target due to its role in the development and progression of a range of cancers 1. Recent clinical data indicate that it is overexpressed in 15-20% of breast cancers 2, 3 and it has long been associated with more aggressive mammary tumours and poorer patient prognosis 4. HER2 promotes cancer cell proliferation, invasion, metastasis and angiogenesis via activation of the PI3K/Akt and Ras/Raf/MEK/ERK signalling cascades 5. Attenuation of oncogenic signalling pathways is regulated by endocytosis 6 but HER2 has been shown to be confined to plasma membrane protrusions and is termed an 'endocytosis-resistant' receptor 6, 7. Aspects regarding its endocytosis are controversial: there is evidence that HER2 expression impairs clathrin coated pit formation at the plasma membrane 8, that the receptor constitutively recycles 9, 10 and that the HER2 cytoplasmic domain contains a membrane retention signal 11. Recycling pathways also maintain the plasma membrane pool of HER2, enabling repeated stimulation and prolonged signalling. HER2 is the preferred dimerisation partner of all ErbB family members and upon heterodimerisation, it confers downregulation resistance to partner receptors. Thus, endocytosis-deficient, HER2-containing heterodimers elicit prolonged downstream signalling responses 12, 13.

HER2 has been therapeutically targeted by the monoclonal antibody trastuzumab (Tz). Despite being an approved treatment for almost two decades, the mechanisms underlying Tz activity and resistance are complex and only partially understood. To varying, often limited degrees, Tz has been reported to induce HER2 endocytosis and downregulation 14. It has demonstrated activity against HER2-overexpressing breast cancers but resistance and relapse represent significant problems 15. Recently a number of strategies have been published to address this, directly or indirectly based on the mechanism of receptor crosslinking. We, and others, have published that inducing crosslinking/clustering of HER2 with targeted antibodies leads to enhanced lysosomal delivery and degradation 16-21. Other therapeutic strategies exploiting the endocytosis potentiating effect of receptor clustering include dendrimers, aptamers and nanoparticles 22-24.

Here we show, compared with Tz alone, induction of Tz crosslinking in HER2‑overexpressing breast cancer cells promotes extensive plasma membrane remodelling, driving internalisation and delivery of antibody:receptor complexes to lysosomes. HER2 degradation is accompanied by activation of MEK and ERK with concomitant downregulation of HER3, but not EGFR. The crosslinking approach, however, is shown to only be effective in Tz-sensitive cell lines and not in a model of acquired Tz-resistance. This has significant implications for existing and future antibody-based cancer therapies that seek to exploit receptor-crosslinking for enhanced lysosomal delivery and degradation.

## Materials and Methods

### Cell lines and culture

SKBR3 (ER^-^, HER2^+^), BT474 (ER^+^, HER2^+^) and BT474 clone 5 (ER^+^, HER2^+^, Tz resistant) and MCF7 (ER^+^, HER2^-^) human breast cancer cells were obtained directly from LCG Standards, UK supplier of authenticated American Type Culture Collection (ATCC) cells. The ATCC and RRID codes for the cell lines are as follows: SKBR3 (ATCC: HTB-30, RRID: CVCL_0033), BT474 (ATCC: HTB-20, RRID: CVCL_0179), BT474 clone 5 (ATCC: CRL-3247, RRID: CVCL_AQ07) and MCF-7 (ATCC: HTB-22, RRID: CVCL_0031). All cells were cultured at 37°C in a 5% CO_2_ humidified atmosphere in complete medium: Dulbecco's Modified Eagle Medium (DMEM, Gibco, Fisher, UK) containing phenol red and supplemented with 10% v/v foetal bovine serum (FBS, Gibco, Fisher, UK), referred to as complete medium. Cell lines were used to 28 passages (maximum) and were subject to quarterly mycoplasma testing (MP0035, Sigma).

### Generation and characterisation of fluorescent, biotinylated trastuzumab construct

Tz solution (containing: 21mg/ml Tz, L-histidine HCl, L-histidine, α,α-trehalose dehydrate, and polysorbate) was donated by Velindre Cancer Centre (Cardiff, UK). For labelling, as previously published 16, this clinical formulation (1.5 mL, 32 mg Tz) was sequentially buffer-exchanged into phosphate buffered saline (PBS) pH 7.4, via two 10 mL Zeba Spin desalting columns (Fisher Scientific). Of the 1.7 mL eluted from the column, 1.5 mL of Tz (30 mg) was added directly to 1 mg NHS-Alexa647 (Life Technologies) and 400 µl of 1 mg/mL NHS-biotin (Fisher Scientific) PBS (pH 7.4). Conjugation reactions were allowed to proceed for 1 hr at room temperature. Resulting Tz-construct was purified using two 10 mL Zeba Spin desalting columns (1 mL/column) in PBS (pH 7.4). This was then sterile filtered (0.2 µm), aliquoted into Eppendorf tubes, snap-frozen and stored at -20 °C.

UV-visible absorbance spectrum of the Tz-construct was obtained using a Jasco V-650 UV-Vis spectrophotometer. Biotin concentrations were calculated using an HABA Biotin Quantification Kit according to manufacturer's instructions (Pierce, UK) 16.

### Antibody-receptor crosslinking targeting HER2

Cells were seeded into 6-well plates (for Western blotting) or 35 mm glass bottomed imaging dishes (for confocal microscopy) to obtain 70-80% confluency on the day of experimentation (~60 hr post-seeding). For crosslinking: cells were incubated for 30 min at 37 °C, 5% CO_2_ with 50 nM Tz-construct in 500 µL complete imaging medium (CIM: phenol-red free DMEM supplemented with 10% FBS). Cells were washed three times with sterile PBS (pH 7.4) and then incubated with 500 µl of 1 μg/mL streptavidin (SA) in CIM or CIM only for 1 hr. The cells were then washed 3x in PBS before being incubated in complete medium for 6 hr (standard crosslinking protocol) or as indicated in the text.

### siRNA depletion of endocytic proteins

*siRNA sequences*: Dharmacon ON-TARGET plus SMARTpool siRNAs were obtained from GE Healthcare targeting AP2M1 (L-008170-00-0005), CAV-1 (L-003467-00-0005) and FLOT-1 (L‑010636-00-0005). Non-targeting siRNA control, GFP (5'-GGCUACGUCCAGGAGCGCAdTdT-3') was synthesised by MWG.

SKBR3 cells (300,000) were seeded in 35 mm glass- bottomed imaging dishes (MatTek) and incubated for 24 hr in complete medium. The transfection mixture was prepared the following day by mixing: transfection reagent 2.4 μL Dharmafect1 (Fisher, UK) with 237.6 μL OptiMEM (Fisher, UK) and incubating at room temperature for 5 min. Meanwhile 12 μL of 5 μM siRNA was mixed with 228 μL OptiMEM. Diluted Dharmafect1 was then mixed with the diluted siRNA and incubated at room temperature for 30 min. The cell medium (in the 6-well plate) was replaced with 1920 μL of compete medium. The transfection mixture (480 μL) was then added to each well, giving a 25 nM final siRNA concentration. The cells were incubated in their transfection medium at 37°C, 5% CO_2_ for 48 hr before further experimentation.

### Western blotting

Cell lysates were collected on ice from 6-well plates by scraping with NP40 lysis buffer supplemented with protease and phosphatase inhibitors (Roche). Denatured/reduced samples (18 μg total protein/ sample) were separated by SDS-PAGE (Bio-Rad) and were transferred to Polyvinylidene fluoride (PVDF) membranes. Sample membranes were blocked in 5% milk in PBST (0.025% Tween20) for 1 hr at room temperature then incubated overnight at 4 °C with primary antibodies diluted in 2% milk in PBST (see Supplementary Table 1). Membranes were washed 3x 5 min in PBST prior to 1 hr incubation with species specific secondary antibodies (all from Peirce) diluted 1:1000 in 2% milk in PBST. An exception to this was for anti-tubulin HRP conjugate, for which the signal was detected after washing. Following secondary antibody incubation, membranes were washed 3x 5 min in PBST ready for signal detection by enhanced chemiluminescence (ECL, Femto, Fisher). Membranes were imaged using a ChemiDoc XRS+ (Bio-Rad, Hemel Hempstead, UK) running ImageLab with images obtained before saturation is reached. Densitometry was performed with Fiji software and normalised to loading control bands for relevant total/housekeeping proteins.

### Antibody stripping for membrane reprobing

Where required e.g. for comparing levels of activated and total protein, membranes were stripped and re‑probed as follows: 2x 5 min washes in PBS followed by 2x 5 min washes in dH_2_O, they were stripped for 10 min in 0.2 M NaOH, washed twice 2x 5 min in dH_2_0 and washed a final 2x 5 min in PBS. Stripped membranes were then used for immunoblotting, following the procedure from the blocking step onwards.

### Dextran labelling of lysosomes for co-localisation analysis

Lysosome co-localisation analysis was performed as previously published 16. Briefly: cells were pulsed for 16 hr with the fluid-phase endocytic probe Dextran-555 (D34679, Molecular Probes) 25 diluted to 200 μg/mL in 500 µL complete medium. This was subsequently chased over the duration of the crosslinking experiment with dextran-free complete imaging medium.

### Actin disruption and visualisation

SKBR3 cells (120,000) were seeded onto glass coverslips and cultured at 37°C, 5% CO_2_ for 48 hr. Tz uptake experiments (+/- crosslinking) were then performed in the continuous presence of DMSO diluent control or 5 µM CytD to disrupt the actin cytoskeleton 26. At the end of the uptake experiment (total CytD/vehicle exposure time = 7.5 hr) cells were fixed in 3% PFA for 15 min, washed 3x with PBS then permeabilised in 0.2% Triton X-100 and washed a further 3x in PBS. To visualise the filamentous actin the cells were incubated with 1.0 µg/ml Rhodamine- Phalloidin in PBS for 15 min at room temperature. The cells were finally washed 3x with PBS then dipped once into PBS, once into distilled water and coverslips were then mounted in 12 µL DAKO oil on glass microscope slides and imaged by confocal microscopy.

### MEK/ERK inhibition

SKBR3 cells (300,000) were seeded in 35 mm glass-bottomed imaging dishes (MatTek) and cultured at 37°C, 5% CO_2_ for 48 hr. Tz uptake (+/- crosslinking) was then performed in the continuous presence of DMSO diluent control or 20 nM trametinib (total trametinib/vehicle exposure time = 7.5 hr). Cells were then imaged live by confocal microscopy.

### Live-cell imaging confocal microscopy

Cells were imaged live with a Leica SP5 confocal laser-scanning microscope equipped with a 37°C heated stage CO_2_ perfused humidified chamber (Ibidi), 488 nm Argon, 543/633 nm Helium-Neon lasers and a 63× 1.4 numerical aperture (NA) oil immersion objective. Gain and offset settings were optimised for the untreated/control cells at the start of each independent experiment and were maintained for the duration. Images were captured with the sequential scanning mode using a line average of 2.

### Scanning electron microscopy (SEM)

SKBR3 cells (125,000) were seeded onto glass coverslips in a 12-well plate. After 24 hr incubation (37°C, 5% CO_2_) the cells were incubated for 30 min with 50 nM Tz-bi-647 or diluent control in imaging medium. They were then washed 3x in PBS and incubated for 10 min with either 1 μg/mL SA (or diluent control) in imaging medium. At room temperature: the cells were washed 3x in PBS, fixed in 2% glutaraldehyde for 30 min, washed a further 3x in PBS and post-fixed in 1% Osmium tetroxide in PBS for 30 min. The cells were washed another 3x in PBS then dehydrated through sequential, 10 min incubations with increasing concentrations of ethanol from 50%-100%. Dehydrated cells were then transferred from ethanol to hexamethyldisilazane (HMDS) by 10 min washes in Ethanol:HMDS solutions starting at a ratio of 50:50 and repeated with 25:75 10:90 and ending on three 10 min incubations in 100% HDMS. Excess HMDS was aspirated and the cells were allowed air dry overnight in a fume cupboard. Coverslips were mounted onto stage inserts and were sputter-coated with gold-palladium (BIO‑RAD SC‑500 for sputter coating with argon gas for plasma) before imaging by SEM (Zeiss Sigma HD Field Emission Gun Analytical SEM). For imaging the accelerating voltage (EHT) was 5.00 kV with probe current of <100 pA and a working distance of 5 mm. Images showing the SKBR3 cell surface had a magnification of 10.00 K X and 30.00 K X.

### Cell viability

Cell viability at 72 hr post-crosslinking treatment was assessed by CellTiter-Blue^®^ assay (#G8080, Promega) according to manufacturer's instructions. Briefly, cells were seeded (3,000 per well) into black 96-well plates and incubated at 37°C, 5% CO_2_ overnight. The following day the crosslinking protocol was carried out and cells were returned to the incubator for 67 hr after the final medium change. At 68 hr post-crosslinking, 20 μL of CellTiter-Blue reagent was added to each well and the plates were returned to the incubator for 4 hr. Viable cell- generated fluorescence was quantified with a Fluostar Optima fluorescent plate reader (544Ex/590Em).

### Statistical methods

For qualitative experiments e.g. microscopy data (Fig. 2E, 3A, 4, 5A, 6A) at least two independent experiments were carried out. For quantitative experiments e.g. all immunoblotting and co-localisation analysis, three independent experiments were performed. Where duplicates were obtained in immunoblotting data, separate means were calculated for each independent experiment. Quantitative data are presented as the average of the independent means ± standard error of the mean (SE). One-way ANOVA tests were performed to analyse each dataset across the three independent experiments. Where statistically significant differences were obtained (P<0.05) Tukey Post-hoc testing was performed to determine significant mean differences between groups. In the main body of text only Tukey p-values are reported, for full ANOVA statistic outputs see Supplementary Figure S6.

## Results

### Tz:HER2 crosslinking induces concomitant downregulation of HER3 but not EGFR

We have previously shown that SA induced crosslinking of biotinylated Tz can enhance endocytic delivery and degradation of HER2 in lysosomes 16. We used our previously published methods to label Tz with biotin and Alexa647, producing a fluorescent antibody amenable to crosslinking. Construct characterisation by UV spectral analysis and biotin quantification assay indicated means of 3.9 fluorophores and 6.0 biotin moieties per antibody (Supplementary Figure S1A). For functional characterisation, the Tz-construct was applied to HER2-overexpressing (SKBR3 and BT474) cells and HER2^-/low^ (MCF-7) cells followed by SA to induce crosslinking (or diluent control). Cells were imaged live by confocal microscopy at 1 and 7 hr post- crosslinking Figure S1B). At 1 hr, bright labelling was detected at the plasma membrane of the SKBR3 and BT474 (HER2-overexpressing) cells with minimal evidence of binding in the MCF-7 (HER2^-/low^) cells; indicating specificity for HER2. At 7 hr, SA-induced crosslinking lead to greater Tz internalisation in SKBR3 and BT474 cells: as demonstrated by reduced plasma membrane labelling and increased vesicular fluorescence. No differences +/- crosslinking were detected in the MCF-7 cells and collectively the results indicated the construct behaved as previously described (16.

**Figure 1:**
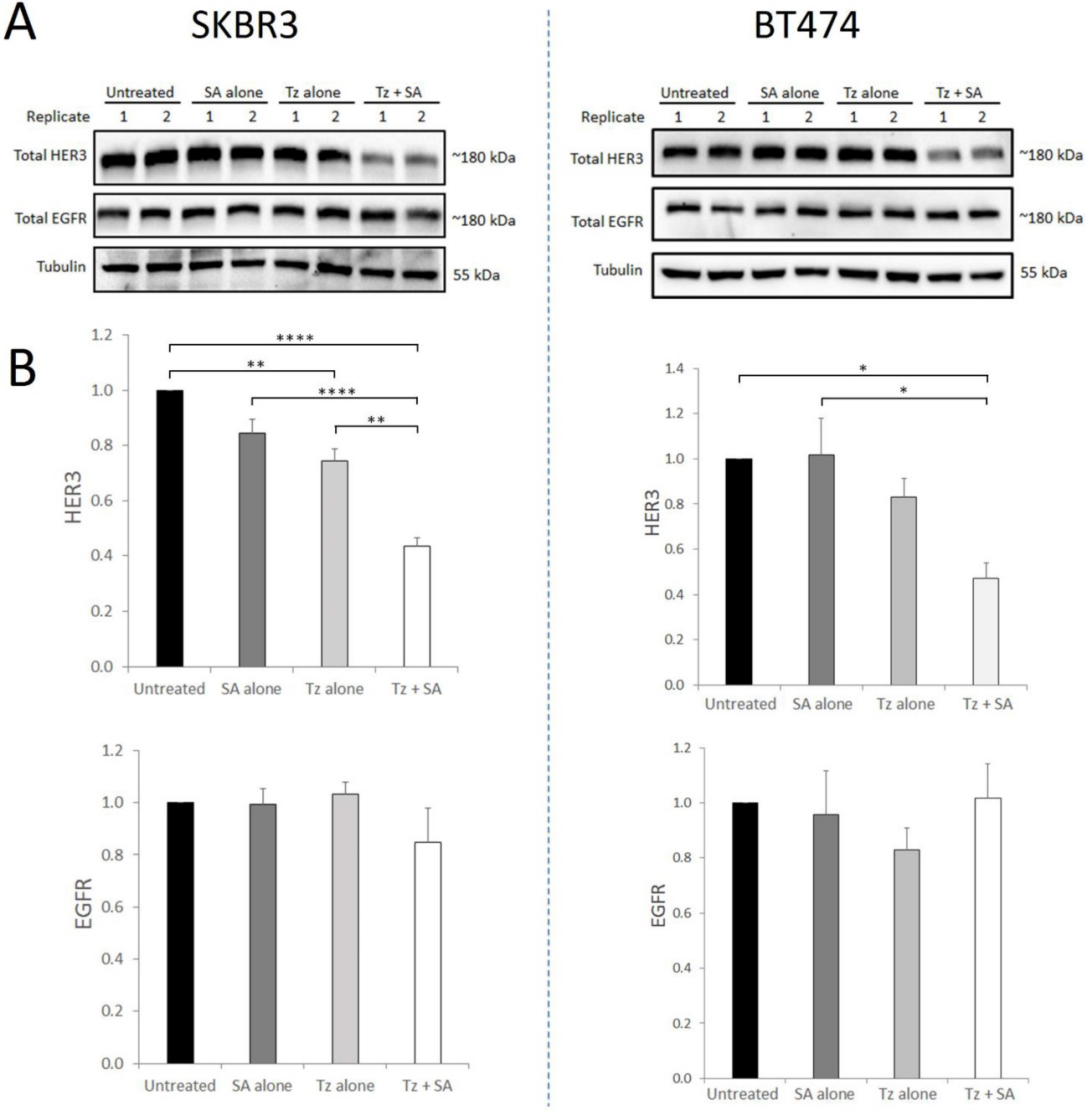
** Crosslinking-specific reduction in HER3 in HER2^+^ breast cancer cells is induced by Tz:HER2-crosslinking**. SKBR3 and BT474 cells were either untreated (control) or incubated with Tz diluent control 30 min followed by SA alone for 1 hr, Tz alone for 30 min (+ 1 hr SA diluent control), or Tz for 30 min followed by SA for 1 hr (Tz+SA). Following treatments the cells were chased in CIM for 6 hr. Cell lysates were collected from three independent experiments A) Western blotting was performed for HER3, EGFR and β-tubulin *(representative blot shown)* and b) band intensities were quantified using ImageJ software. Mean from 3 independent experiments shown, error bars depict SE, *p≤0.05, **p≤0.01, ****p≤0.0001. **The data revealed that HER3, but not EGFR, was specifically reduced by HER2 targeted crosslinking.**

HER2 readily dimerises with other ErbB receptors, forming heterodimers at the cell membrane with ligand-bound partners. We therefore sought to determine whether induction of Tz:HER2 crosslinking, that we have shown to potentiate HER2 degradation 16, could also affect the receptor's binding partners. For this we performed crosslinking in SKBR3 and BT474 cells and (at 7 hr) examined HER3 and EGFR levels. Uncrosslinked Tz in SKBR3 cells produced a small but statistically significant reduction in HER3 levels compared to control with no change in EGFR levels (Figure 1). In BT474 cells uncrosslinked Tz did not significantly alter levels of either receptor. In both cell lines Tz-crosslinking induced consistent, substantial and significant reductions in HER3 compared with controls while EGFR levels were not significantly altered. HER3 and EGFR were shown to co-precipitate with HER2 in both SKBR3 and BT474 cell lines (Supplementary Figure S2) indicating that both proteins share an association with HER2 in these cells.

### Tz-HER2 crosslinking alters HER2's activation and downstream signalling profile

**Figure 2:**
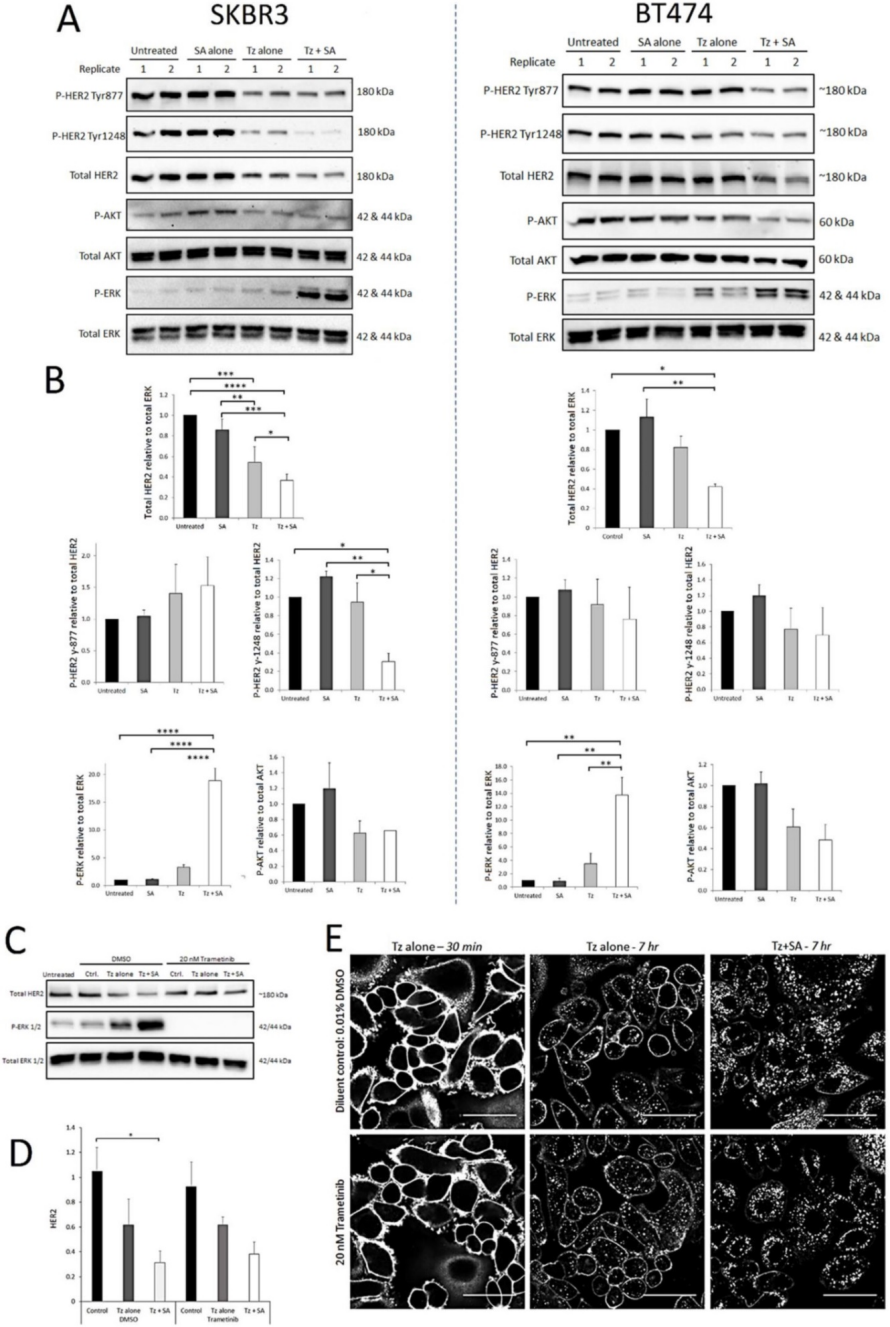
** Crosslinking-specific alterations in HER2 levels, activation and downstream signalling are detected in HER2^+^ breast cancer cells following induction of Tz:HER2-crosslinking and the enhanced ERK activation is a consequence not a driver of crosslinking enhanced Tz:HER2 endocytosis**. SKBR3 cells and BT474 cells were either untreated (control) or incubated with Tz diluent control 30 min followed by SA alone for 1 hr, Tz alone for 30 min (+ 1 hr SA diluent control), or Tz for 30 min followed by SA for 1 hr (Tz+SA) then chased for a further 6 hr in CIM. Cell lysates were collected from three independent experiments, representative blots are shown. A) Western blotting was performed for HER2, pHER2 (Tyr-1248), P-AKT (Ser-473), total AKT, P-ERK (Thr-202/Tyr-204) and total ERK. B) Band intensities were quantified using ImageJ software. Mean from 3 independent experiments is shown, error bars represent SE, *p≤0.05, **p≤0.01, ***p≤0.001, ****p≤0.0001. **The data demonstrate that crosslinking reduced levels of HER2 and induced activation of ERK in both cell lines and reduced the proportion of HER2 phosphorylated at Tyr-1248 in SKBR3 cells.** C) SKBR3 cells were, in the continuous presence of 20 nM trametinib or diluent control, either untreated (control) or incubated with Tz diluent control 30 min followed by SA alone for 1 hr, Tz alone for 30 min (+ 1 hr SA diluent control), or Tz for 30 min followed by SA for 1 hr (Tz+SA) followed by a 6 hr chase in CIM. D) Band intensities were quantified using ImageJ software. Mean from 3 independent experiments is shown, error bars represent SE, *p≤0.05. **Data show that there was no significant difference in crosslinking-enhanced downregulation of HER2 in the presence of MEK/ERK inhibition.** E) Confocal microscopy analysis of ERK activation dependency of crosslinked Tz:HER2 internalisation. Cells were incubated, in the presence of 20 nM trametinib or diluent control, with Tz for 30 min and imaged (left column). Cells were then incubated for 1 hr with either diluent control (middle column) or SA (right column). Following treatments cells were chased in complete imaging medium for 6 hr and imaged. *Scale bar = 50 µm.*** Data demonstrate that inhibiting MEK/ERK did not significantly alter crosslinking-enhanced internalisation of Tz.**

Activated HER2 heterodimers stimulate oncogenic signalling via the PI3K/Akt and Ras/Raf/ MEK/ERK pathways. Tz, as a synthetic HER2 ligand, affects receptor activation and downstream signalling differently depending on dose, duration of exposure and therapeutic sensitivity/resistance 27-29. We therefore aimed to determine whether crosslinking altered the activation profile of HER2 at selected phosphorylation residues and/or if the activities of critical downstream signalling nodes Akt and ERK were affected. From Western blots, levels of activated protein were calculated relative to total levels of the same protein (e.g. P-Akt to total Akt) except total HER2 which was normalised to total ERK (housekeeping/loading control). As we reported previously 16 compared with Tz alone, crosslinking (Tz+SA) caused greater downregulation of HER2 from untreated control levels in both SKBR3 and BT474 cells (Figure 2). Levels of Tyr-877 P-HER2 were reduced in proportion to HER2 for both BT474 and SKBR3 cells. This was also true of Tyr-1248 P-HER2 in the BT474 cell line. However, in SKBR3 cells there was a significantly greater reduction in Tyr‑1248 P‑HER2 relative to total HER2 induced by crosslinked Tz compared with Tz alone (Figure 2B). Akt activation was generally reduced by Tz and Tz+SA, as has been reported for Tz previously 30, however, there was no difference in the reduction with or without crosslinking. Tz alone did not significantly induce ERK activation above baseline, however, there was a marked, crosslinking-specific increase in ERK activation in both cell lines.

SKBR3 cells were assessed for ERK activation and total HER2 levels at earlier time points (1 and 3 hr) post-crosslinking (Supplementary Figure S3A and B). The data demonstrate that initially (1 hr) ERK was strongly phosphorylated following treatment with Tz ± crosslinking. At both time points ERK appeared to be more activated in Tz+SA treated cells compared with Tz alone but differences did not reach significance. After 1 hr, total HER2 levels were not significantly different with and without Tz and/or crosslinking but at 3 hr there was an indication that Tz+SA treatment had begun to reduce the receptor levels. The combined data from Figures 2 and S2 suggest that in a 7 hr period crosslinking gradually reduces the levels of HER2 while amplifying/ prolonging Tz-induced ERK activation.

Various pathways can stimulate ERK activation and we analysed the phosphorylation status of the upstream ERK kinase (MEK) in response to crosslinking. Data from SKBR3 and BT474 lysates suggested that a proportion of the ERK phosphorylation seen in Figure 2 was a result of increased MEK activity (Supplementary Figure S3 C-D). We sought to determine whether this MEK/ERK activity was a consequence of, or a requirement for, crosslinking- enhanced Tz:HER2 endocytic degradation. For this we conducted antibody uptake and receptor downregulation experiments in the presence and absence of the MEK inhibitor trametinib. Figure 2C demonstrates efficient abolition of ERK activity in trametinib treated cells and coupled with Figure 2D indicates that this inhibition did not significantly alter the HER2 downregulating effect of crosslinking. This is supported by confocal microscopy data (Figure 2E) demonstrating that the inhibition of MEK/ERK did not affect the ability of crosslinking to increase Tz internalisation. Reduced membrane labelling and increased vesicular accumulation of Tz in the crosslinking treated cells occurred with and without trametinib.

### Tz:HER2 crosslinking induced endocytosis occurs by clathrin dependent and independent endocytic pathways

**Figure 3:**
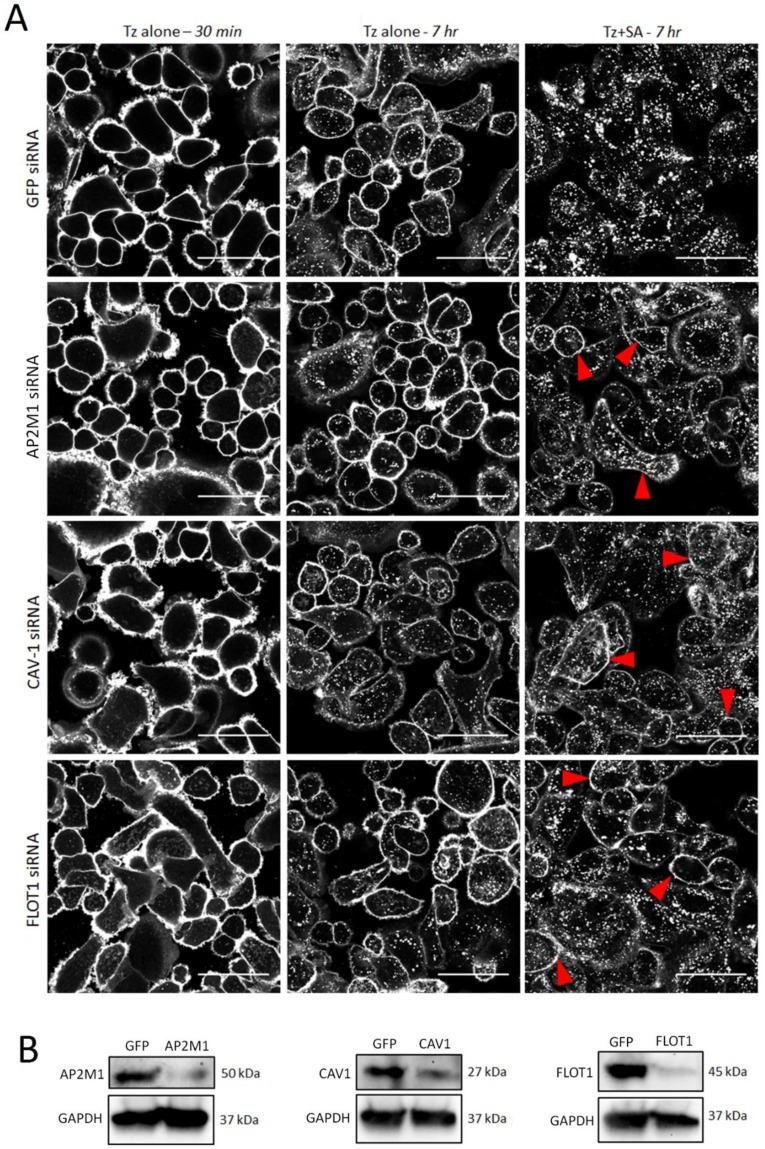
** Enhanced uptake of Tz:HER2 induced by crosslinking is reduced by inhibiting clathrin dependent and independent endocytosis.** A) Confocal microscopy analysis of endocytic pathways governing internalisation of Tz-HER2 membrane clusters. SKBR3 cells were transfected with GFP (control) siRNA or sequences targeting AP2M1, caveolin-1 (CAV1) or flotillin-1 (FLOT1) to impair clathrin-dependent and -independent endocytic pathways respectively. Transfected cells (48 hr) were incubated with Tz for 30 min and imaged (left column). Cells were then incubated for 1 hr with either diluent control (middle column) or streptavidin (SA, right column). Following treatments cells were chased in complete imaging medium for 6 hr and imaged. Scale bar = 50 µm, red arrows depict increased membrane retention of Tz. Data show that SA induced crosslinking promoted HER2 internalisation compared with diluent controls under all conditions, however, in AP2M1, CAV1 and FLOT1 depleted cells clustering-induced internalisation was significantly reduced compared with controls. B) Western blotting control data using lysates of the cells imaged in (A). Immunoblots show successful depletion of AP2M1, CAV1 and FLOT1.

Clathrin dependent and independent mechanisms have been implicated in the endocytosis of HER2 and Tz 8, 31, 32 and in crosslinking-based approaches for enhanced receptor internalisation 21. To investigate the contribution of specific pathways to crosslinking induced Tz:HER2 endocytosis we used siRNA to deplete critical endocytic targets 33. SKBR3 cells were transfected with control siRNA or those targeting AP2M1 to impair clathrin-dependent endocytosis or caveolin-1 (CAV1) or flotillin-1 (FLOT1) to inhibit selected clathrin-independent pathways. After 48 hr transfection, cells were incubated with Tz for 30 min and imaged, then incubated for 7 hr with either diluent control or SA and imaged (Figure 3A). SA-induced crosslinking promoted HER2 internalisation compared with diluent controls under all conditions, however, in AP2M1, CAV1 and FLOT1 depleted cells crosslinking-induced internalisation was reduced compared with controls. This was demonstrated by increased Tz plasma membrane retention at and reduced vesicular accumulation in the crosslinking treated, endocytosis compromised cell models compared with control crosslinking-treated cells. Lysates made from these cells after imaging were used to confirmed depletion of target proteins by Western blotting (Figure 3B).

### Tz-HER2 crosslinking induces remodelling of the plasma membrane

**Figure 4:**
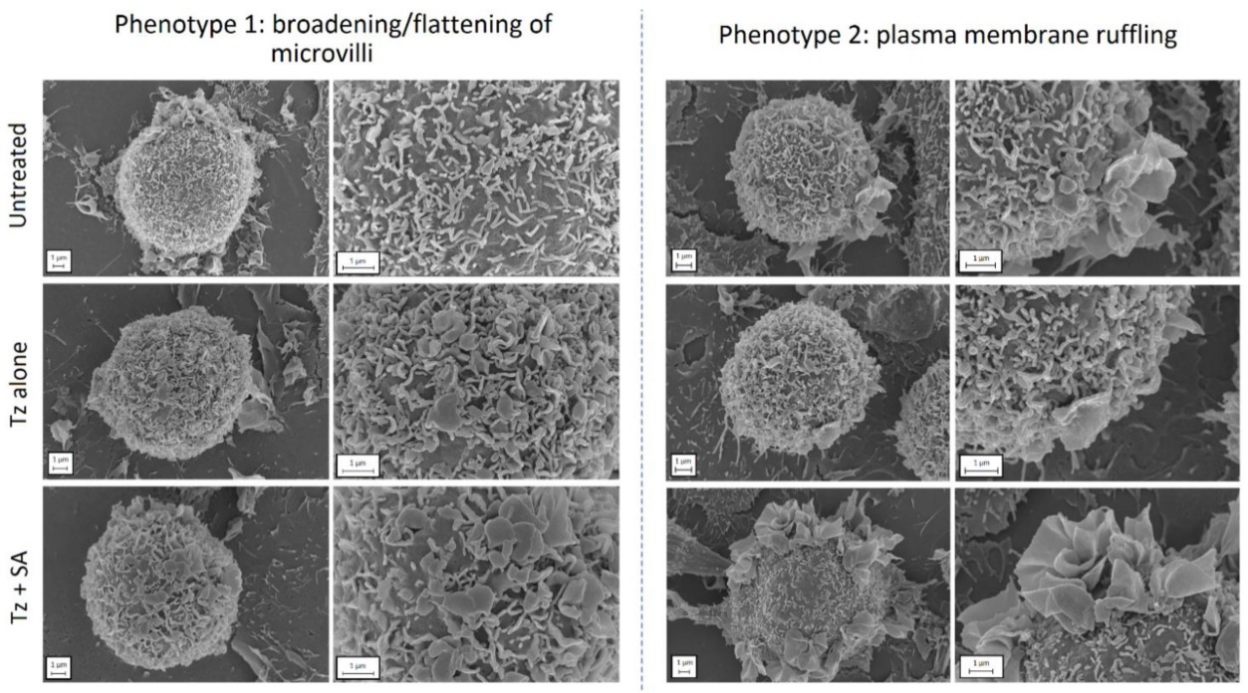
** Crosslinking induces remodelling at the plasma membrane of SKBR3 cells.** Cells were either untreated (control) or incubated with Tz alone for 30 min (+ 10 min SA diluent control), or Tz for 30 min followed by SA for 10 min (Tz+SA). Cells were fixed, dehydrated, sputter coated and imaged by SEM. Two cell phenotypes were observed in response to crosslinking (bottom row): 1) broadened/flattened microvilli and 2) plasma membrane ruffles indicative of macropinocytosis*. Scale bar = 1 µm.*

To investigate the cellular processes preceding endocytosis in more detail, we examined the structure/topography of the plasma membrane at an early time point post-crosslinking. For this we prepared samples of control, Tz treated and Tz+SA treated SKBR3 cells for SEM, fixing cells after 30 min of Tz addition and 10 min +/- SA crosslinking. Untreated cells were also examined as controls and micrographs display the microvilli that typically cover the surface of SKBR3 cells (Figure 4). This is a feature that has been previously reported for HER2-overexpressing cells: HER2 has both been shown to be retained in these microvilli 7 and to induce their formation 34. The application of Tz alone induced some remodelling at the plasma membrane typified by flattening and broadening of the microvilli, this phenotype was however greatly enhanced in the SA-crosslinked cells. A secondary phenotype was also observed following crosslinking in the form of extensive plasma membrane ruffling located on one or more regions of the cells.

### Tz:HER2 endocytosis, with and without crosslinking, is actin-dependent

**Figure 5:**
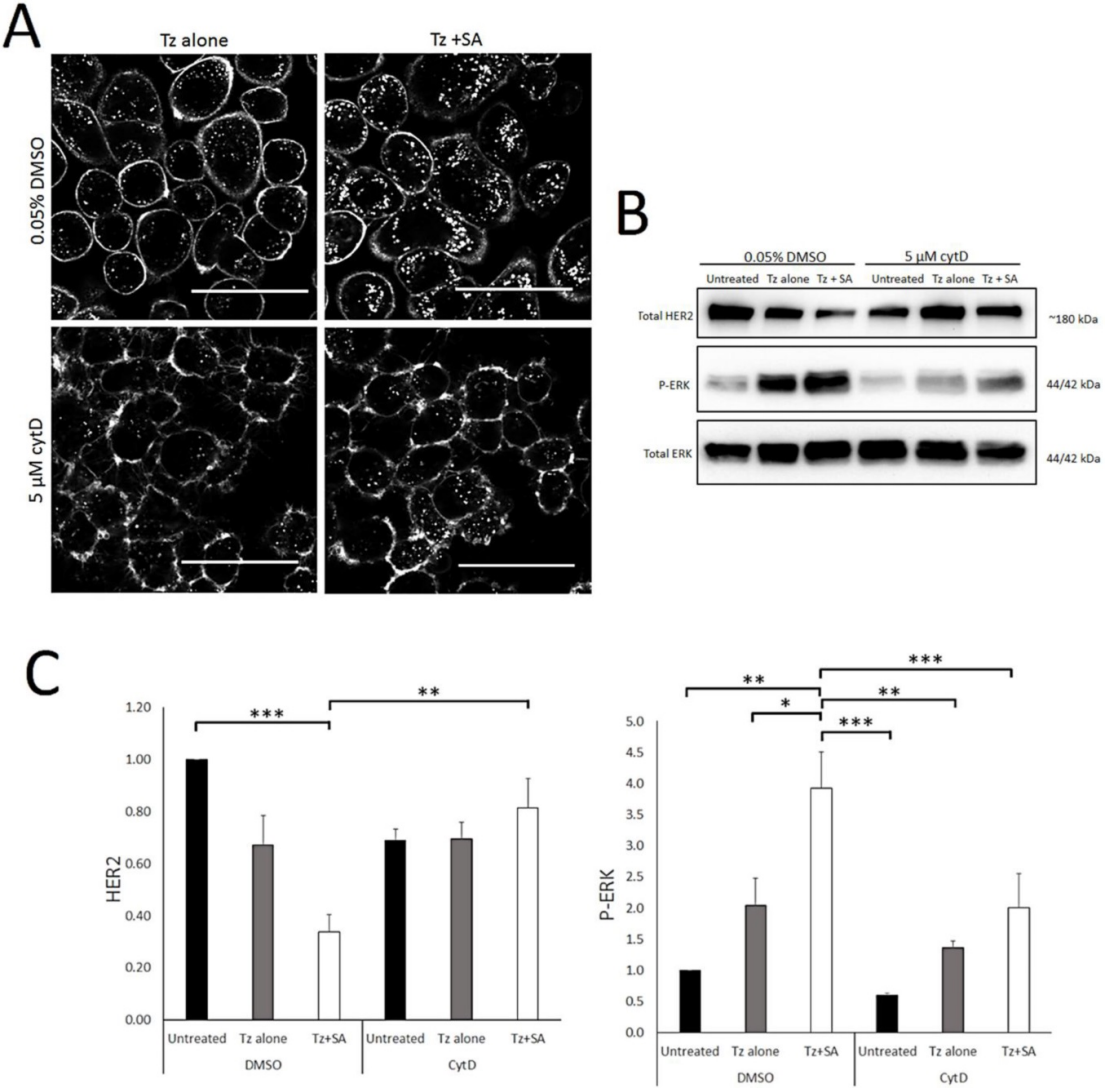
** Tz internalisation and crosslinking-enhanced downregulation of HER2 is actin dependent.** A) Confocal microscopy analysis of actin dependency of crosslinked Tz:HER2 internalisation. SKBR3 cells were incubated, in the presence of 5 µM CytD or diluent control, with Tz for 30 min. They were then incubated for 1 hr with either diluent control (middle column) or SA (right column) ± CytD. Following treatments cells were chased in complete imaging medium (containing CytD or diluent control) for 6 hr and imaged. *Scale bar = 50 µm.*** Data demonstrate that disruption of filamentous actin inhibited internalisation of Tz and prevented crosslinking from enhancing Tz endocytosis.** B) SKBR3 cells were, in the continuous presence of 5 µM CytD or diluent control, either untreated (control) or incubated with Tz diluent control 30 min followed by SA alone for 1 hr, Tz alone for 30 min (+ 1 hr SA diluent control), or Tz for 30 min followed by SA for 1 hr (Tz+SA). C) Band intensities were quantified using ImageJ software. Mean from 3 independent experiments is shown, error bars represent SE, *p≤0.05, **p≤0.01, ***p≤0.001. **Data show that disruption of filamentous actin prevented crosslinking-induced downregulation of HER2 and restricted ERK activation.**

Actin is a critical regulator of endocytosis, plasma membrane organisation and structure 26, 35. Other HER2-crosslinking based approaches have suggested a role for actin/macropinocytosis in HER2 internalisation 21 and this combined with our SEM data led us to examine the actin-dependency of crosslinking. For this we conducted control versus Tz alone versus Tz+SA experiments in the presence and absence of cytochalasin D (CytD). CytD is an actin filament disrupting agent and widely-used inhibitor of macropinocytosis 26. Confocal microscopy data in Figure 5A depicts SKBR3 cells incubated with Tz alone or Tz+SA in the continuous presence of CytD or DMSO diluent control. Crosslinking, as expected, increased vesicular accumulation and reduced plasma membrane localisation of Tz in control cells. However, in the presence of CytD this effect was dramatically reduced: with or without SA crosslinking Tz was largely confined to the plasma membrane. These findings were supported by Western blotting data examining levels of HER2 and P-ERK (Figure 5B). CytD inhibited crosslinking- induced downregulation of HER2 and reduced receptor levels. Surprisingly, ERK activation was reduced by CytD treatment though the trend of Tz induced activation and enhancement by crosslinking was still evident despite being reduced in magnitude. Control data in Supplementary Figure S4A demonstrates via rhodamine-phalloidin labelling that actin is disrupted under these experimental conditions.

### Crosslinking does not enhance Tz-HER2 endocytosis and degradation in a cell model of acquired Tz resistance

**Figure 6:**
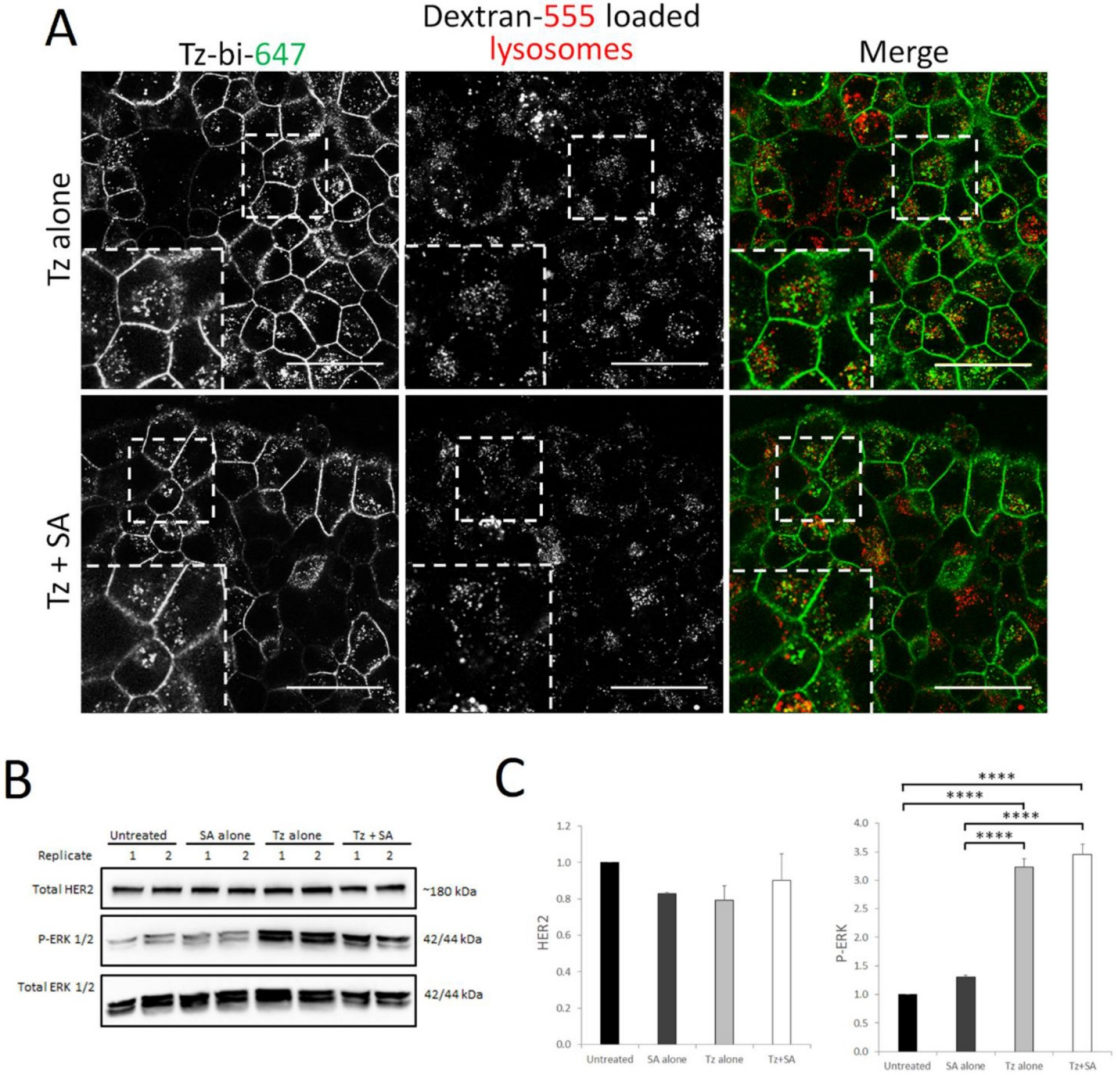
** Crosslinking does not enhance lysosomal targeting of Tz in a Tz-resistant breast cancer cell line and consequently does not induce HER2 downregulation or ERK activation.** BT474 clone 5 (acquired Tz resistant) cells were either untreated (control) or incubated with Tz diluent control 30 min followed by SA alone for 1 hr, Tz alone for 30 min (+ 1 hr SA diluent control), or Tz for 30 min followed by SA for 1 hr (Tz+SA) then a 6 hr chase in CIM. For microscopy experiments lysosomes were labelled by dextran pulse-chase prior to antibody treatment. A) Confocal microscopy showing no significant difference in colocalisation between Tz and lysosomes ± SA crosslinking. B) Western blotting was performed for HER2, P-ERK and total ERK *(representative blot shown)* and C) band intensities were quantified using ImageJ software. Mean from 3 independent experiments is shown, error bars represent SE, ****p≤0.0001. **Data revealed no difference in HER2 downregulation or ERK activation in Tz-resistant cells treated with Tz alone or Tz+SA crosslinking.**

Therapeutic resistance represents a significant limitation of Tz treatment and a number of clustering based approaches are being developed to address this clinical problem 18, 21, 36. We therefore tested our crosslinking strategy in the BT474 clone 5 model of acquired Tz resistance. This cell line has been shown to express surface levels of HER2 comparable to the parental BT474 cells 37 and we were able to confirm that our Tz-construct could still bind to the resistant clone (Supplementary Figure S4B). We initially investigated whether crosslinking would enhance endocytic uptake and lysosomal delivery of Tz:HER2 as we demonstrate here and previously in the parental BT474 line 16. For this we pre-labelled late endolysosomes with dextran prior to crosslinking and subsequent co-localisation analysis by confocal microscopy. To our surprise, data in Figure 6A demonstrate that there was little colocalisation between Tz and the late endolysosomes with or without crosslinking in the Tz-resistant cell line. We then examined the levels of HER2 and ERK activation status in response to Tz and crosslinking treatments (Figure 6B-C). In contrast to our crosslinking findings in Tz-sensitive cells (Figure 2) we found that in the resistant clone there were no significant differences in HER2 levels in response to Tz or crosslinking treatment. ERK was phosphorylated in response to Tz alone and to Tz+SA, however there was no enhancement of ERK activation by crosslinking.

Cell TiterBlue experiments performed at 72 hr post-crosslinking in the BT474 clone 5, BT474 and SKBR3 cells demonstrated no significant effects of the 30 min Tz alone treatment or of Tz followed by SA (Supplementary Figure S5A). This was unsurprising as we have previously showed, and again demonstrate in Supplementary Figure S5B that HER2 levels recover within 48 hr of Tz crosslinking challenge 16.

## Discussion

Elucidating the fundamental molecular mechanisms governing antibody receptor crosslinking in cancer cells may improve existing therapeutic strategies, guide the development of future approaches and provide insight into overcoming treatment resistance. Here we sought to characterise the mechanisms involved in a crosslinking strategy targeting HER2: an approach that has been directly and indirectly attempted with diverse nanomedicines including nanoparticles, dendrimers and therapeutic antibodies 18, 23, 24.

The endocytosis resistance of HER2 is well documented 6 and in line with this we observed relatively slow receptor downregulation in response to Tz treatment. It was 7 hr before HER2 was significantly depleted by Tz ± crosslinking. This represents a relatively low rate of endocytic downregulation compared with a prototypical receptor such as ligand- bound EGFR where the majority of the receptor degrades within 1 hr of epidermal growth factor (EGF) stimulation 38. There are conflicting reports in the literature about the ability of Tz to drive lysosomal degradation of HER2 6 but our data adds to the body of evidence that the antibody can induce receptor downregulation. We show, in line with our previous findings 16 and with the literature 19, that crosslinking of HER2 at the plasma membrane can enhance the endocytic degradation of HER2 in Tz-sensitive breast cancer cell lines. We show here for the first time that ERK activation is a product of HER2-crosslinking but is not required for internalisation. A previous study using polyclonal antibodies against HER2 showed higher levels of P‑ERK but the effect was not explored in the report 20. Thus, ERK activation appears to be a specific and reproducible consequence of HER2 crosslinking, independently of the approach used. It may in fact be a broader consequence of plasma-membrane protein crosslinking as it has been reported in response to a diverse range of crosslinked targets including integrins, and EGFR 39, 40.

From a clinical perspective hyperactivation of ERK may be considered undesirable as it has been linked to the development of Tz-induced toxicity. Phosphorylation of ERK has been documented in cardiomyocytes following Tz treatment, this inhibited autophagy and stimulated reactive oxygen species (ROS) production leading to cardiomyopathy 41. Importantly, ERK hyperactivation is also a known Tz resistance mechanism 42. Since we have previously 16, and here, demonstrated HER2 recovery within 48 hr of Tz or crosslinking treatment it may be that the ERK activation is a compensatory signal to the cell to produce more HER2 in response to receptor depletion. This could occur via stimulation of the ETS transcription factor ER81 which is known to be activated by ERK and to regulate HER2 promoter activity 43. The recovery of HER2 represents a limitation of a crosslinking based approach as a monotherapy for driving HER2 downregulation. On the other hand, HER2 replenishment at the plasma membrane is potentially beneficial for enhancing ADC treatment as it would enable retargeting should a cell survive an initial ADC dose.

The difference between cell lines after crosslinking in the proportion of Tyr‑1248 P-HER2 relative to total HER2 may relate to differences in the levels of other ErbB receptors and in HER2 dimerisation profile. Such differences are known between SKBR3 and BT474 cells and have been reported to modulate Tz sensitivity 44. A number of possible scenarios could account for the reduced Tyr-1248 P-HER2 in SKBR3 cells, it may be that crosslinking inhibits HER2 activation at this residue, or that crosslinking promotes phosphatase activity against Tyr-1248 P-HER2. Alternatively, it may be that a greater proportion of Tyr-1248 P-HER2 is degraded in crosslinking treated cells compared with Tz alone. The finding might be considered particularly surprising given that phosphorylation of this tyrosine residue is known to couple HER2 to the Ras/Raf/MEK/ERK pathway 45, which we show here to be more, not less, activated by crosslinking. Phosphorylation of HER2 and ERK have been shown to be reduced with longer term Tz exposure (>24 hr) following an initial spike in activation with short term (1 hr) exposure 46. We can speculate that crosslinking may alter the kinetics of the Tz response in breast cancer cells, altering the magnitude and duration of the HER2 and ERK signals induced by the antibody alone. For ERK in particular this may have significant cellular implications as the size and duration of this protein's activation are known to be critical determinants of signalling outcome 47. Irrespective of mechanism, reduced Tyr-1248 P-HER2 levels supports the therapeutic potential of crosslinking, as phosphorylation of this residue has been linked to poor clinical outcome 48.

We show here for the first time that HER3 is concomitantly downregulated with HER2 in response to crosslinking while EGFR levels are unchanged. It can, in part, be explained by HER2's documented propensity to preferentially dimerise with HER3 (over other ErbB receptors) 12. Tz alone was previously shown to induce HER3 downregulation though only to a limited degree and over a three day period of continuous incubation 45. Here we demonstrate significant potentiation of HER3 downregulation by Tz crosslinking. We suggest this may be the result of crosslinked Tz being able to induce more widespread downregulation of HER3:HER2 heterodimers than can be achieved with Tz alone. This we base on the principle that Tz alone can only induce large-scale crosslinking of HER2 homodimers whereas the multivalent SA enables extensive oligomerisation of HER2 homodimers and HER2:HER3 heterodimers (Supplementary figure S7). Interestingly, targeting of HER3 with polyclonal antibodies has recently been shown to induce downregulation of HER3 and HER2 49 which, with our findings, suggests that crosslinking of either receptor allows concomitant targeting its dimerisation partner. The HER2:HER3 heterodimer is a potent, constitutive inducer of oncogenic signalling and HER3 transcriptional upregulation is a known mechanism of resistance to HER2 inhibition 50. Breast cancers driven by HER2 homodimers have been shown to be more sensitive to Tz 27 so crosslinking may offer a therapeutic advantage by enabling targeting of tumours driven by HER2:HER3 heterodimers.

Our siRNA depletion studies showing partial inhibition of crosslinking enhanced Tz endocytosis in CME and CIE impaired cells, indicate that multiple endocytic pathways are involved in the process. This provides a therapeutic advantage as degeneracy of uptake mechanism potentially subverts resistance mechanisms involving perturbations of specific endocytic pathways such as those documented for Tz and T-DM1 resistance 31, 51. The involvement of multiple endocytic pathways and of actin to HER2 endocytosis has been suggested previously using chemical inhibitors and dynamin mutants to impede uptake of multiple HER-targeted antibodies 21. Our data, obtained with more-specific siRNAs, more clearly implicate caveolin and flotillin in the processes governing crosslinking-enhanced Tz:HER2 endocytosis.

Actin filaments (stress fibres) have been shown to attach at sites of clustered plasma membrane receptors, including those crosslinked by antibody binding, and this is thought to facilitate endocytosis 52. Actin inhibition, here and in the literature for a HER2-targeted antibody mixture 21, has been shown to inhibit antibody:receptor internalisation. This has been interpreted as macropinocytosis being the primary uptake mechanism of Tz. However, the specificity of CytD for macropinocytosis is questionable owing to actin's varied role in receptor endocytosis 53. We therefore favour the description of our findings as evidence of actin dependency rather than of macropinocytosis. We suggest that actin may play a role in the changes in plasma membrane organisation/structure, seen with Tz and exacerbated by crosslinking. Plasma membrane remodelling, including actin reorganisation, has been reported for antibodies targeting EGFR 54. However, for these antibodies uptake was shown to occur via circular dorsal ruffles and the experiments were conducted under serum-starvation conditions. Crucially, our experiments are conducted in full-serum which better reflects physiological conditions and provides greater confidence for *in vitro - in vivo* translation. Actin reorganisation is a complex, highly regulated process so there may be further mechanistic insight to be gained from future studies dissecting critical actin regulators such as Rho, Rac and PAK1 55.

Our SEM images show the microvilli-rich plasma membrane of HER2 overexpressing cells in very high detail. Previous studies have demonstrated HER2 enrichment in membrane protrusions 7 and this work was extended by others who showed the microvilli were directly generated by HER2 overexpression and clustering 34. The later study showed by total internal reflection microscopy (TIRFM) that antibody binding to HER2 can deform microvilli, an effect our SEM work shows topographically and we show for the first time that plasma membrane remodelling can be dramatically enhanced by crosslinking. Distortion of the HER2 extracellular domain by DARPins has been shown to alter receptor activation and signalling 56 and we hypothesise that crosslinking by Tz at the extracellular juxtamembrane domain, may induce similar changes in HER2 structure and organisation. This would directly couple the morphological changes in HER2-containing microvilli to the alterations in HER2 activity and downstream signalling.

Ado-trastuzumab emtansine (T‑DM1/Kadcyla) is an ADC that was developed to improve Tz efficacy by using it to deliver the cytotoxic emtansine into HER2-overexpressing cells^57^. However, T-DM1 has a fairly poor cost:benefit profile compared with Tz alone and is only used in patients who have developed Tz resistance. HER2's endocytic deficiency significantly limits the efficacy of standard ADC approaches as lysosomal delivery is required for intracellular cytotoxic release. Our discovery that crosslinking was unable to enhance Tz internalisation and HER2 downregulation in a cell model of Tz-resistance provides invaluable information with respect to understanding the limitations of the approach in future therapies. This is particularly pertinent given the increasing evidence of resistance to T-DM1 developing 51, 57 and in the current trend towards crosslinking in HER2 targeting. In a study using a biparatopic antibody targeting distinct HER2 epitopes to crosslink and downregulate the receptor, the construct was shown to have activity against cell models of *de novo* Tz resistance 18. Taken with our findings in an *acquired* Tz-resistant cell line the data might be pointing to critical differences in the mechanisms driving primary and secondary resistance and this may have implications for therapy selection (personalised medicine). The results may simply reflect a difference in the crosslinking strategy itself e.g. single agent (biparatopic antibody) versus sequential SA-mediated crosslinking. Further experimentation, beyond the scope of this study, is required to determine to what extent the sensitivity of a HER2-overexpressing cell line to a crosslinking strategy depends on a particular resistance mechanism or cell type. The role of endocytosis in Tz sensitivity and in HER2 crosslinking susceptibility should particularly be explored.

## Conclusions

Our studies have demonstrated that crosslinking significantly enhances the endocytosis of Tz in HER2-overexpressing breast cancer and induces effects of potential therapeutic benefit including promotion of HER2 and HER3 downregulation and alteration of HER2 activation and signalling. The improved endocytic capacity and lysosomal targeting of crosslinked HER2 would theoretically translate to T-DM1, improving the efficacy of this therapeutic currently limited in clinical use. We have revealed that a cell model of acquired Tz-resistance is not responsive to crosslinking with respects to HER2 endocytosis, signalling and downregulation. This has implications for existing therapeutic approaches based on antibody crosslinking (e.g. combined Tz and pertuzumab) as well as future strategies seeking to tackle Tz-resistance. Overall, our results will aid the design of future Tz-based therapeutics with improved capacity for HER2 downregulation and cytotoxic delivery. Our findings provide a new perspective on the crosslinking-based receptor targeting in breast cancer. They may also have implications for the whole remit of antibody and crosslinking-based receptor targeting in cancer.

## Supplementary Material

Supplementary methods, figures, and table.

## Abbreviations

ADC: Antibody drug conjugate
AP2M1: Adaptor protein complex 2 subunit mu
CAV1: Caveolin 1
CIM: Complete imaging media
CytD: Cytochalasin D
DMEM: Dulbecco's modified eagle medium
EHT: Extra high tension voltage level
EGF: Epidermal growth factor
EGFR: Epidermal growth factor receptor
ERK: Extracellular signal related kinase
HDMS: Hexamethyldisilazane
HER2/3: Human epidermal growth factor receptor 2/3
FLOT1: Flotillin 1
PBS: Phosphate buffered saline
PVDF: Polyvinylidene fluoride
NA: Numerical aperture
SA: Streptavidin
SEM: Scanning electron microscopy
T-DM1: Ado-trastuzumab emtansine
TIRFM: Total internal reflection microscopy
Tz: Trastuzumab
Tz-bi-647: Biotinylated trastuzumab conjugated to Alexa647
