# Supplementary material for: Strategic Trastuzumab Mediated Crosslinking Driving Concomitant HER2 and HER3 Endocytosis and Degradation in Breast Cancer
Source: J Cancer. 2020 Mar 5;11(11):3288–302. doi: 10.7150/jca.32470 (PMC7097966; doi:10.7150/jca.32470)
Supplement: Supplementary file 1 — Supplementary methods, figures, and table. [file jcav11p3288s1.pdf]

SUPPLEMENTARY INFORMATION

Supplementary Methods:

Immunoprecipitation of EGFR and HER3 following HER2 pull down in SKBr3, BT474 and BT474 Clone 5

The three cell lines were grown to 80% confluency and treated with trastuzamab or diluent control plus or minus streptavidin following the crosslinking method outlined in the main paper. After a 15 min streptavidin treatment the cells were incubated with 100 µL lysis buffer (1% Triton X100, 20 mM Tris-HCl, 150 mM NaCl, pH 7.5, and miniComplete protease inhibitors). Cell lysate protein content was assessed using BCA assay after being centrifuged at 13,000 x *g* for 10 min at 4 °C and the supernatant collected. Immunoprecipitation was performed using published protocol (58) adapted and outlined below.

Generation of IP beads: anti-HER2 mAb 7C2 (BSA/glycerol free) was purchased from Insight Biotechnology (Wembley, UK) and diluted to 400 µg/mL in 0.4 M Sodium Citrate, 0.1M Sodium HEPES, pH7.5. All 100 µg 7C2 was added to 5 mg Ultralink Biosupport (Fisher Scientific) and allowed to react for a minimum of 3 hr at room temperature under constant rotation. 7C2 beads were pelleted at 1,200 x *g* for 5 min and the supernatant removed. To both 7C2 beads and 5 mg control beads 10X the bead swell volume (10xSV) of 2M glycine in PBS was added and the solution allowed to react for 3 hr at room temperature under rotation. 7C2 and control beads were washed by pelleting (1,200 x *g*, 5 min) the beads and resuspended in 10xSV PBS for 15 min at room temperature under rotation. This was followed by further washes in 10xSV 1M NaCl, and two 10xSV PBS washes. The beads were finally resuspended in 200 µL PBS and kept at 4°C for a maximum of 24 hr.

Immunoprecipitation using Ultralink Biosupport beads: 100 µg of cell lysate was incubated with 15 µL of 7C2 or control beads in a final volume of 200 µL lysis buffer overnight at 4 °C under constant rotation. The beads were pelleted at 1,200 x *g* for 5 min and the pellet was washed twice with 500 µL lysis buffer. The final pellet was resuspended in 15 µL PBS prior to adding SDS loading buffer containing DTT (5 µL). The sample was boiled for 4 min at 96 °C before being separated on a 7.5% SDS-PAGE gel, transferred to PDVF membranes and probed for EGFR, HER3 and finally HER2 as per the method section.

Supplementary Figures:

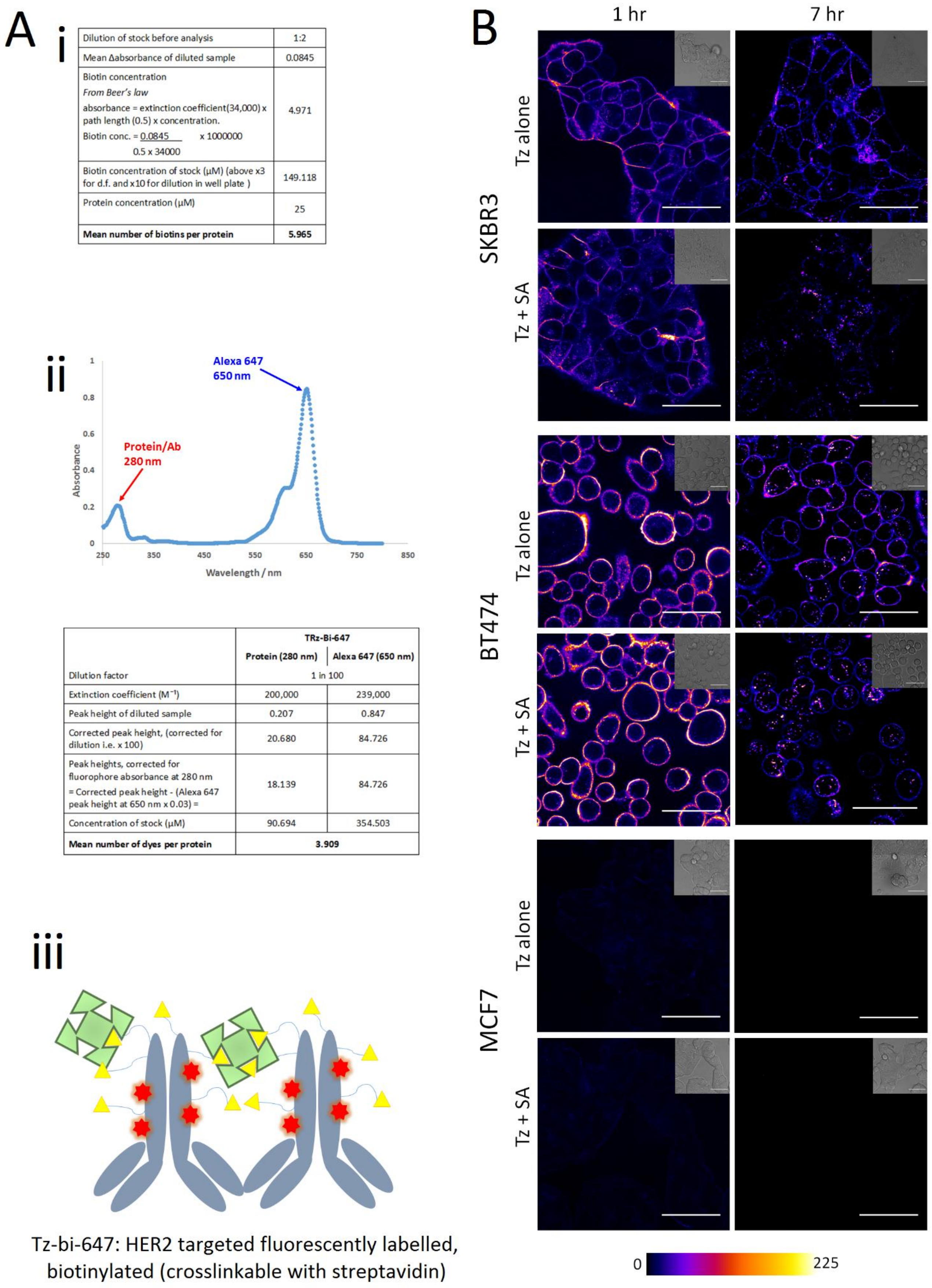

Dilution of stock before analysis	1:2
Mean Δabsorbance of diluted sample	0.0845
Biotin concentration<br>*From Beer's law*<br>absorbance = extinction coefficient(34,000) x path length (0.5) x concentration.<br>Biotin conc. = $\frac{0.0845}{0.5 \times 34000} \times 1000000$	4.971
Biotin concentration of stock (μM) (above x3 for d.f. and x10 for dilution in well plate)	149.118
Protein concentration (μM)	25
**Mean number of biotins per protein**	**5.965**

	**TRz-Bi-647**	
	**Protein (280 nm)**	**Alexa 647 (650 nm)**
Dilution factor	1 in 100	
Extinction coefficient (M^{-1})	200,000	239,000
Peak height of diluted sample	0.207	0.847
Corrected peak height, (corrected for dilution i.e. x 100)	20.680	84.726
Peak heights, corrected for fluorophore absorbance at 280 nm<br>= Corrected peak height - (Alexa 647 peak height at 650 nm x 0.03) =	18.139	84.726
Concentration of stock (μM)	90.694	354.503
**Mean number of dyes per protein**	**3.909**	

SUPPLEMENTARY FIGURE S1: Characterisation of Tz-bi-647. A) chemical Tz-bi-647 characterisation: (i) results and analysis of biotinylation assay demonstrating an average of 6.0 biotin moieties per antibody. (ii) UV spectral analysis showing an average of 3.9 fluorophores per antibody. (iii) schematic illustrating how biotinylated Tz can be crosslinked by SA. B) Tz-bi-647 cellular uptake and crosslinking-induced endocytosis characterisation. SKBR3, BT474 and MDA-MB-231 cells were incubated with Tz for 30 min then incubated for 1 hr with either diluent control (left column) or SA (right column). Following treatments the cells were chased in complete imaging medium for 6 hr and imaged. *Scale bar = 50 µm.* **Data demonstrate that crosslinking enhances Tz internalisation in HER2$^+$ SKBR3 and BT474 cells and not in HER2$^-$ MDA-MB-231 cells where relatively little Tz bound and was endocytosed.**

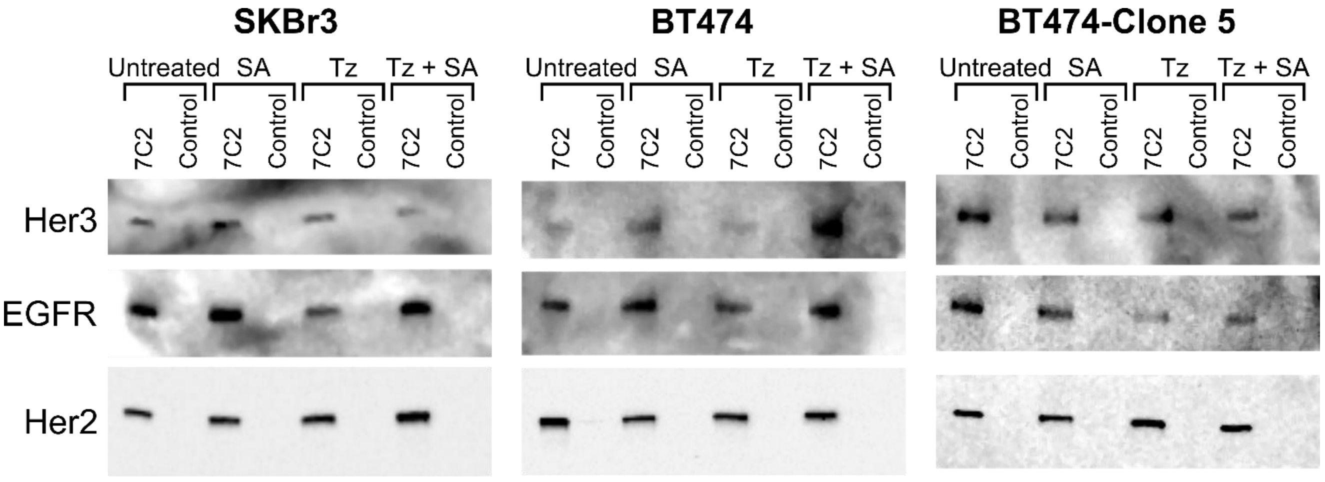

SUPPLEMENTARY FIGURE S2: HER3 and EGFR co-precipitates with HER2 following immunoprecipitation in SKBR3, BT474 and BT474 clone 5. Cells were incubated with trastuzumab or diluent control for 30 min followed by SA (or diluent control) for 15 min under tissue culture conditions. Cells were lysed and HER2 was precipitated through an overnight incubation of 100 µg of lysate with beads linked to an anti-HER2 Ab. HER3, EGFR and HER2 levels were assessed by western blot. **The data show that both EGFR and HER3 a co-precipitated following immunoprecipitation of HER2.**

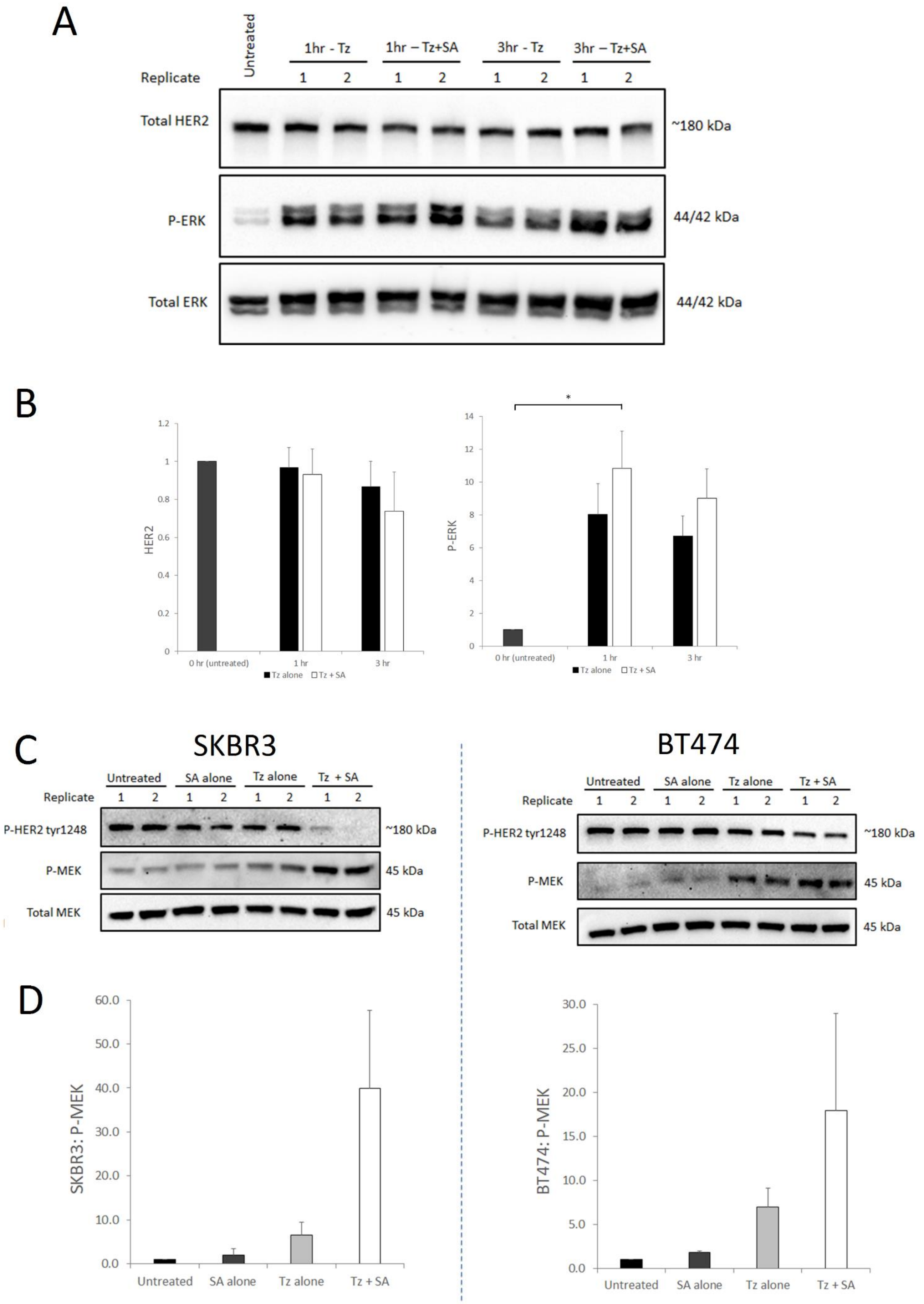
A
Untreated
1hr - Tz
1hr – Tz+SA
3hr - Tz
3hr – Tz+SA
Replicate
1 2 1 2 1 2 1 2
Total HER2
~180 kDa
P-ERK
44/42 kDa
Total ERK
44/42 kDa
B
HER2
1.2
1
0.8
0.6
0.4
0.2
0
0 hr (untreated)
1 hr
3 hr
Tz alone
Tz + SA
*
P-ERK
14
12
10
8
6
4
2
0
0 hr (untreated)
1 hr
3 hr
Tz alone
Tz + SA
C
SKBR3
Untreated
SA alone
Tz alone
Tz + SA
Replicate
1 2 1 2 1 2 1 2
P-HER2 tyr1248
~180 kDa
P-MEK
45 kDa
Total MEK
45 kDa
BT474
Untreated
SA alone
Tz alone
Tz + SA
Replicate
1 2 1 2 1 2 1 2
P-HER2 tyr1248
~180 kDa
P-MEK
45 kDa
Total MEK
45 kDa
D
SKBR3: P-MEK
60.0
50.0
40.0
30.0
20.0
10.0
0.0
Untreated
SA alone
Tz alone
Tz + SA
BT474: P-MEK
30.0
25.0
20.0
15.0
10.0
5.0
0.0
Untreated
SA alone
Tz alone
Tz + SA

SUPPLEMENTARY FIGURE S3: Early-time point profile of ERK-activation and HER2 levels in crosslinking treated breast cancer cells and evidence of MEK activation following crosslinking in breast cancer cells. A) SKBR3 cells were either untreated (control) or incubated with Tz diluent control 30 min followed by SA alone for 1 hr, Tz alone for 30 min (+ 1 hr SA diluent control), or Tz for 30 min followed by SA for 1 hr (Tz+SA). For the 3 hr time point cells were subject to a 2 hr chase in CIM before lysate collection. Cell lysates were collected from three independent experiments, representative blots are shown. Western blotting was performed for total HER2, P-ERK (Thr202/Tyr204) and total ERK. B) Band intensities were quantified using ImageJ software. *Mean from 3 independent experiments is shown, error bars represent SE, *p≤0.05.* **Data show ERK activation and slight reduction in HER2 following crosslinking challenge over 3 hr time course, ERK was also activated by HER2 but there was evidence of this beginning to subside.** C) SKBR3 cells and BT474 cells were either untreated (control) or incubated with Tz diluent control 30 min followed by SA alone for 1 hr, Tz alone for 30 min (+ 1 hr SA diluent control), or Tz for 30 min followed by SA for 1 hr (Tz+SA) then a 6 hr chase in CIM. Cell lysates were collected from three independent experiments, representative blots are shown. Western blotting was performed for P-HER2 Tyr1248, P-MEK (Ser217/221) and total MEK. D) Band intensities were quantified using ImageJ software and mean from 3 independent experiments is shown (*Error bars represent SEM)*. **The data demonstrate that crosslinking induced MEK activation of varying magnitude.**

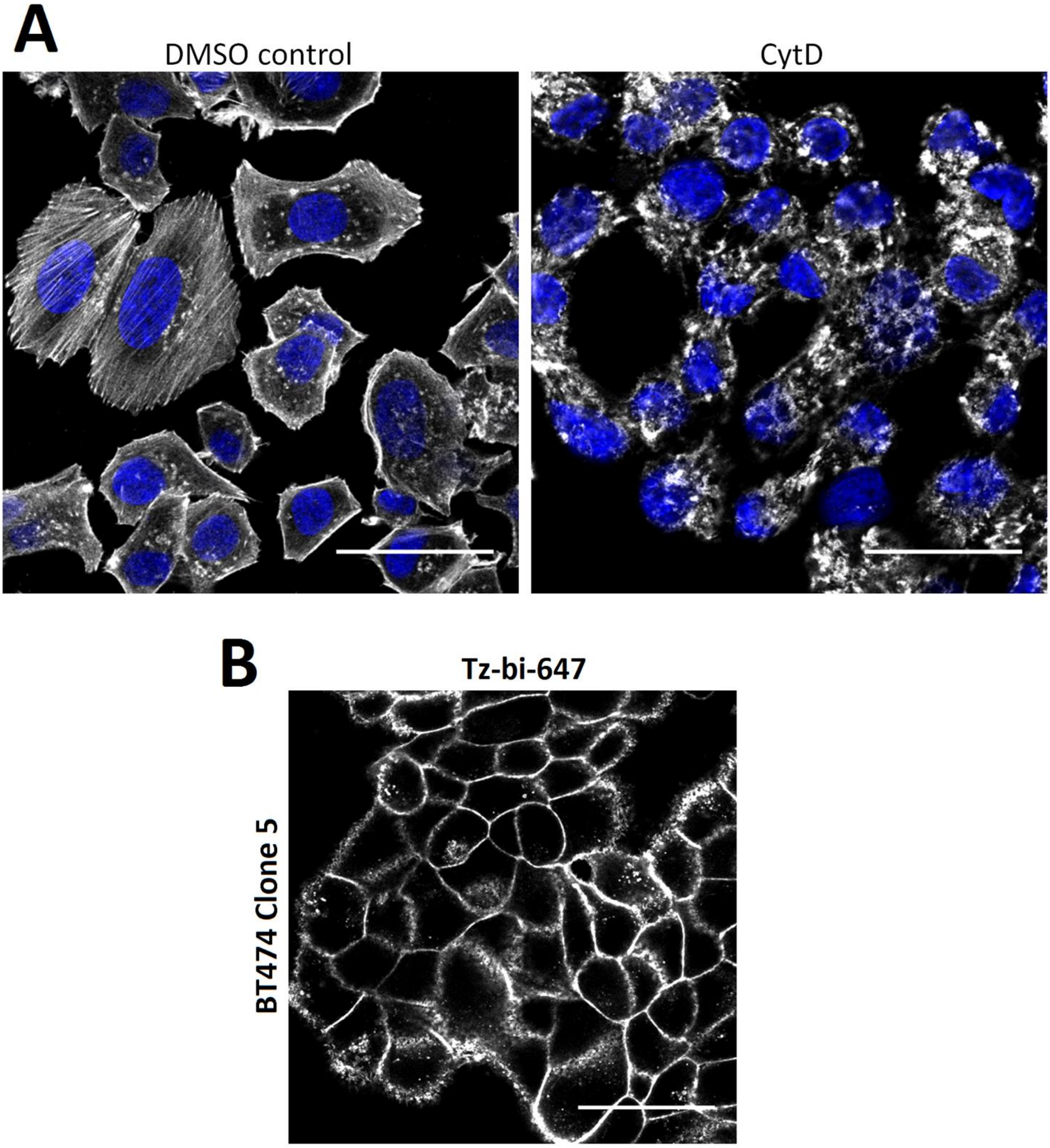

SUPPLEMENTARY FIGURE S4A: CytD disrupts the actin cytoskeleton of cells under the conditions of the crosslinking experiment. SKBR3 cells were incubated with 5 µM CytD or diluent control for 7.5 hr. Following the treatments cells were fixed in 3% PFA and stained with rhodamine-phalloidin (2µg/mL for 15 min), nuclei were counterstained with Hoechst. *Scale bar = 50 µm.* **Data demonstrate disruption of filamentous actin in the CytD treated cells. 4B: Tz-b-647 binds to Tz-resistant BT474 cells.** BT474 clone 5 cells were incubated with Tz for 30 min then imaged by confocal microscopy. *Scale bar = 50 µm.* **Data demonstrate that the Tz-resistant cell line was still able to bind Tz-bi-647 at the plasma membrane.**

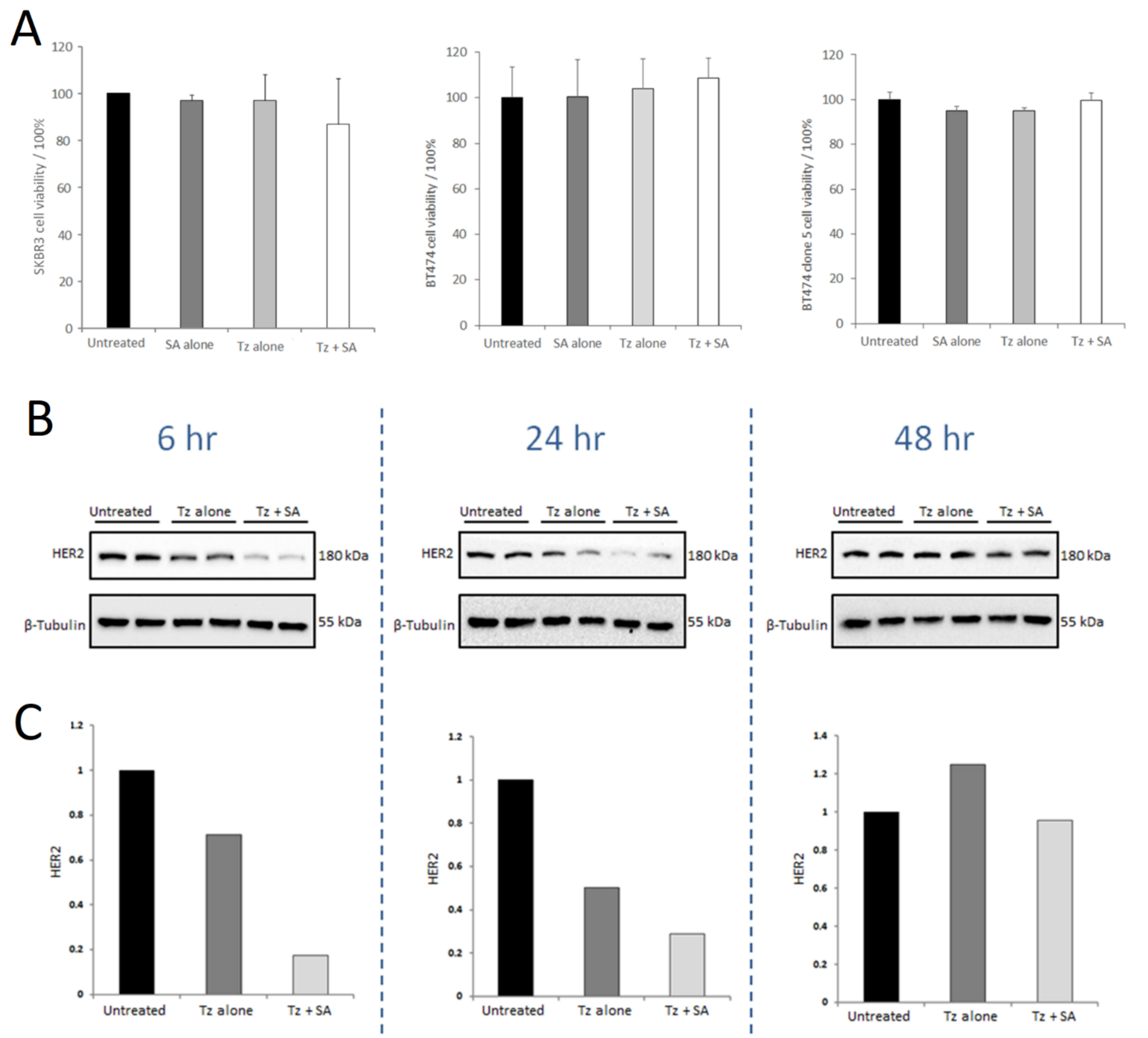

SUPPLEMENTARY FIGURE S5: Breast cancer cell viability is not affected by crosslinking and HER2 levels recover within 48 hr of crosslinking-induced downregulation. A) Viability of SKBR3, BT474 and BT474 clone 5 cells is not significantly altered 72 hr after Avi-Bi-RAC treatment. Cells were either untreated (control) or incubated with Tz diluent control 30 min followed by SA alone for 1 hr, Tz alone for 30 min (+ 1 hr SA diluent control), or Tz for 30 min followed by SA for 1 hr (Tz+SA). Cells were incubated in IM for 72 hr post-crosslinking. Cell Viabilities were analysed by CellTiter- Blue^{TM} assay. Viability was not significantly altered by any of the treatments compared with untreated control cells. B) HER2 levels in Avi-bi-RAC treated SKBR3 breast cancer cells recover in 48 hr, after initial downregulation at 6 and 24 hr post-treatment. SKBR3 cells were either untreated (control) or incubated with Tz alone or Tz followed by SA. The cells were subjected to a post-treatment time course of 6-48 hr in IM. Cell lysates were collected Western blotting was performed for HER2 and β-tubulin. C) Band intensities were quantified using ImageJ software and converted to graphs in Excel. **The data show that Tz:HER2 crosslinking induced by crosslinking treatment at 6 and 24 hr (Tz+SA) reduced HER2 levels compared with Tz alone. By 48 hr the HER2 levels in the Tz and Tz + SA treated cells had increased (recovered).**

A

HER3: SKBR3 cells

F-Statistic	43.4814
Degrees of freedom - Between groups	3
Degrees of freedom - Within groups	8
P-Value	0.0000
**Tukey Post-Hoc Test**	
Untreated vs SA alone	0.0630
Untreated vs Tz alone	0.0047
Untreated vs Tz+SA	0.0000
SA alone vs Tz alone	0.2803
SA alone vs Tz+SA	0.0001
Tz alone vs Tz+SA	0.0014

EGFR: SKBR3 cells

F-Statistic	1.195
Degrees of freedom - Between groups	3
Degrees of freedom - Within groups	8
P-Value	0.3716

HER3: BT474 cells

F-Statistic	6.8356
Degrees of freedom - Between groups	3
Degrees of freedom - Within groups	8
P-Value	0.0134
**Tukey Post-Hoc Test**	
Untreated vs SA alone	0.9988
Untreated vs Tz alone	0.6201
Untreated vs Tz+SA	0.0204
SA alone vs Tz alone	0.5442
SA alone vs Tz+SA	0.0169
Tz alone vs Tz+SA	0.1154

EGFR: BT474 cells

F-Statistic	0.6132
Degrees of freedom - Between groups	3
Degrees of freedom - Within groups	8
P-Value	0.6253

B

HER2: SKBR3 cells

F-Statistic	45.9643
Degrees of freedom - Between groups	3
Degrees of freedom - Within groups	8
P-Value	0.0000
**Tukey Post-Hoc Test**	**P-VALUE**
Untreated vs SA alone	0.0606
Untreated vs Tz alone	0.0003
Untreated vs Tz+SA	0.0000
SA alone vs Tz alone	0.0092
SA alone vs Tz+SA	0.0002
Tz alone vs Tz+SA	0.0409

P-HER2 Tyr1248: SKBR3 cells

F-Statistic	11.8297
Degrees of freedom - Between groups	3
Degrees of freedom - Within groups	8
P-Value	0.0955
**Tukey Post-Hoc Test**	
Untreated vs SA alone	0.5393
Untreated vs Tz alone	0.9863
Untreated vs Tz+SA	0.0115
SA alone vs Tz alone	0.3734
SA alone vs Tz+SA	0.0021
Tz alone vs Tz+SA	0.0179

P-HER2 Tyr 877: SKBR3 cells

F-Statistic	0.6342
Degrees of freedom - Between groups	3
Degrees of freedom - Within groups	8
P-Value	0.6136

P-AKT: SKBR3 cells

F-Statistic	2.2575
Degrees of freedom - Between groups	3
Degrees of freedom - Within groups	8
P-Value	0.1589

P-ERK: SKBR3 cells

F-Statistic	61.3086
Degrees of freedom - Between groups	3
Degrees of freedom - Within groups	8
P-Value	0
Tukey Post-Hoc Test	
Untreated vs SA alone	1
Untreated vs Tz alone	0.5085
Untreated vs Tz+SA	0
SA alone vs Tz alone	0.5261
SA alone vs Tz+SA	0
Tz alone vs Tz+SA	0

HER2: BT474 cells

F-Statistic	8.0401
Degrees of freedom - Between groups	3
Degrees of freedom - Within groups	8
P-Value	0.0085
**Tukey Post-Hoc Test**	**P-VALUE**
Untreated vs SA alone	0.8318
Untreated vs Tz alone	0.6653
Untreated vs Tz+SA	0.0230
SA alone vs Tz alone	0.2617
SA alone vs Tz+SA	0.0075
Tz alone vs Tz+SA	0.1168

P-HER2 Tyr1248: BT474 cells

F-Statistic	0.966
Degrees of freedom - Between groups	3
Degrees of freedom - Within groups	8
P-Value	0.4547

P-HER2 Tyr 877: BT474 cells

F-Statistic	0.3554
Degrees of freedom - Between groups	3
Degrees of freedom - Within groups	8
P-Value	0.7868

P-AKT: BT474 cells

F-Statistic	4.7256
Degrees of freedom - Between groups	3
Degrees of freedom - Within groups	8
P-Value	0.0351
Tukey Post-Hoc Test	
Untreated vs SA alone	0.9998
Untreated vs Tz alone	0.1964
Untreated vs Tz+SA	0.0741
SA alone vs Tz alone	0.1752
SA alone vs Tz+SA	0.0659
Tz alone vs Tz+SA	0.8946

P-ERK: BT474 cells

F-Statistic	15.1871
Degrees of freedom - Between groups	3
Degrees of freedom - Within groups	8
P-Value	0.0011
Tukey Post-Hoc Test	
Untreated vs SA alone	1
Untreated vs Tz alone	0.6789
Untreated vs Tz+SA	0.0019
SA alone vs Tz alone	0.6566
SA alone vs Tz+SA	0.0018
Tz alone vs Tz+SA	0.0073

C

HER2

F-Statistic	3.5874
Degrees of freedom - Between groups	3
Degrees of freedom - Within groups	8
P-Value	0.0324
**Tukey Post-Hoc Test**	
DMSO - untreated vs DMSO - Tz	0.3967
DMSO - untreated vs DMSO - Tz+SA	0.0471
DMSO - untreated vs CytD - untreated	0.9907
DMSO - untreated vs CytD - Tz	0.3985
DMSO - untreated vs CytD - Tz+SA	0.0799
DMSO - Tz vs DMSO - Tz+SA	0.7286
DMSO - Tz vs CytD - untreated	0.7164
DMSO - Tz vs CytD - Tz	0.4068
DMSO - Tz vs CytD - Tz+SA	0.8807
DMSO - Tz+SA vs CytD - untreated	0.1219
DMSO - Tz+SA vs CytD - Tz	0.7266
DMSO - Tz+SA vs CytD - Tz+SA	0.9994
CytD - untreated vs CytD - Tz	0.7184
CytD - untreated vs CytD - Tz+SA	0.1992
CytD - Tz vs CytD - Tz+SA	0.8793

D

HER2

F-value	7.9526
Degrees of freedom - Between groups	3
Degrees of freedom - Within groups	8
P-Value	0.0016
**Tukey Post-Hoc Test**	
DMSO - Untreated vs DMSO - Tz alone	0.0877
DMSO - Untreated vs DMSO - Tz+SA	0.0006
DMSO - Untreated vs CytD - Untreated	0.1141
DMSO - Untreated vs CytD - Tz alone	0.1231
DMSO - Untreated vs CytD Tz+SA	0.5569
DMSO - Tz alone vs DMSO - Tz+SA	0.0813
DMSO - Tz alone vs CytD - Untreated	1.0000
DMSO - Tz alone vs CytD - Tz alone	0.9999
DMSO - Tz alone vs CytD Tz+SA	0.7692
DMSO - Tz+SA vs CytD - Untreated	0.0621
DMSO - Tz+SA vs CytD - Tz alone	0.0574
DMSO - Tz+SA vs CytD Tz+SA	0.0089
CytD - Untreated vs CytD - Tz alone	1.0000
CytD - Untreated vs CytD Tz+SA	0.8477
CytD - Tz alone vs CytD Tz+SA	0.8680

P-ERK

F-value	9.8263
Degrees of freedom - Between groups	3
Degrees of freedom - Within groups	8
P-Value	0.0006
**Tukey Post-Hoc Test**	
DMSO - Untreated vs DMSO - Tz alone	0.4094
DMSO - Untreated vs DMSO - Tz+SA	0.0014
DMSO - Untreated vs CytD - Untreated	0.9681
DMSO - Untreated vs CytD - Tz alone	0.9813
DMSO - Untreated vs CytD Tz+SA	0.4447
DMSO - Tz alone vs DMSO - Tz+SA	0.0366
DMSO - Tz alone vs CytD - Untreated	0.1375
DMSO - Tz alone vs CytD - Tz alone	0.7826
DMSO - Tz alone vs CytD Tz+SA	1.0000
DMSO - Tz+SA vs CytD - Untreated	0.0005
DMSO - Tz+SA vs CytD - Tz alone	0.0042
DMSO - Tz+SA vs CytD Tz+SA	0.0326
CytD - Untreated vs CytD - Tz alone	0.7021
CytD - Untreated vs CytD Tz+SA	0.1531
CytD - Tz alone vs CytD Tz+SA	0.8168

E

HER2

F-Statistic	1.2366
Degrees of freedom - Between groups	3
Degrees of freedom - Within groups	8
P-Value	0.3585

P-ERK

F-Statistic	114.1662
Degrees of freedom - Between groups	3
Degrees of freedom - Within groups	8
P-Value	0.0000
**Tukey Post-Hoc Test**	
Untreated vs SA alone	0.3341
Untreated vs Tz alone	0.0000
Untreated vs Tz+SA	0.0000
SA alone vs Tz alone	0.0000
SA alone vs Tz+SA	0.0000
Tz alone vs Tz+SA	0.5912

Fi

HER2

F-Statistic	0.5923
Degrees of freedom - Between groups	3
Degrees of freedom - Within groups	8
P-Value	0.6761

P-ERK

F-Statistic	4.6177
Degrees of freedom - Between groups	3
Degrees of freedom - Within groups	8
P-Value	0.0227
**Tukey Post-Hoc Test**	
Untreated vs 1 hr Tz alone	0.0999
Untreated vs 1 hr Tz+SA	0.0171
Untreated vs 3 hr Tz alone	0.2696
Untreated vs 3 hr Tz+SA	0.0535
1 hr Tz alone vs 1 hr Tz+SA	0.7813
1 hr Tz alone vs 3 hr Tz alone	0.9554
1 hr Tz alone vs 3 hr Tz+SA	0.9934
1 hr Tz+SA vs 3 hr Tz alone	0.4101
1 hr Tz+SA vs 3 hr Tz+SA	0.9425
3 hr Tz alone vs 3 hr Tz+SA	0.8076

ii

P-MEK: SKBR3 cells

F-Statistic	4.1975
Degrees of freedom - Between groups	3
Degrees of freedom - Within groups	8
P-Value	0.0465
**Tukey Post-Hoc Test**	
Untreated vs SA alone	0.9998
Untreated vs Tz alone	0.972
Untreated vs Tz+SA	0.0627
SA alone vs Tz alone	0.984
SA alone vs Tz+SA	0.0699
Tz alone vs Tz+SA	0.1147

P-MEK: BT474 cells

F-Statistic	1.9415
Degrees of freedom - Between groups	3
Degrees of freedom - Within groups	8
P-Value	0.2015

SUPPLEMENTARY FIGURE S6 Full ANOVA reporting for Western blotting quantification. A) From Figure 1. B) From Figure 2. C) From Figure 3. D) From Figure 6. E) From Figure 7. F) i) From Supplementary Figure S2B, ii Supplementary Figure S2D).

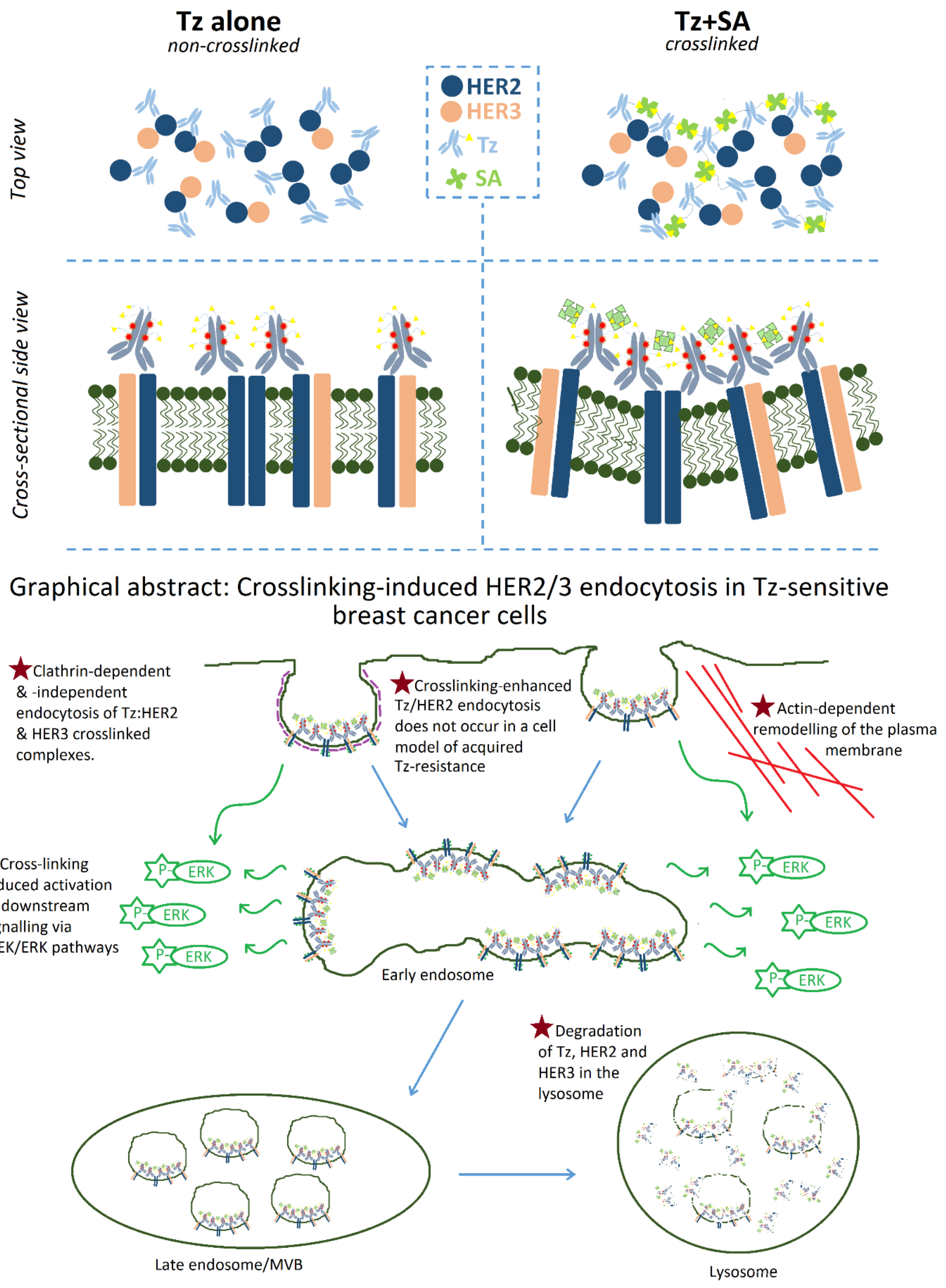

SUPPLEMENTARY FIGURE S7: Graphical hypothesis of Tz:HER2 mediated crosslinking at the plasma membrane. Top view and cross-sectional side view of Tz bound to HER2 at the plasma membrane with and without addition of SA crosslinks. Top row: Tz alone is largely only able to form higher-order crosslinks between HER2 homodimers, HER2:HER3 heterodimers terminate the chain of linkages. Middle row: multivalent SA-crosslinking creates extensive HER2 homodimer and HER2:HER3 heterodimer crosslinkages at the plasma membrane inducing curvature and endocytosis. Bottom section: Tz-crosslinking induces endocytosis, lysosomal trafficking and downregulation of HER2 and HERR3 in Tz-sensitive cells.

Supplementary Table 1: Primary antibodies and dilutions. All antibodies are validated and used according to manufacturer's guidelines or published protocols.

Antibody	Supplier & product number	RRID	Dilution
Mouse—Tubulin HRP	Abcam, ab-21058	AB_727045	1:50,000
Rabbit—HER3	Cell Signalling, #12708	AB_2721919	1:1000
Rabbit—EGFR	Cell Signalling, #2232	AB_10692644	1:1000
Rabbit—HER2	Cell Signalling, #2242	AB_823466	1:1000
Rabbit—P-HER2 tyr-877	Cell Signalling, #2241	AB_2099407	1:500
Rabbit—P-HER2 tyr-1248	Cell Signalling, #2247	AB_331725	1:500
Rabbit—P-Akt ser-243	Cell Signalling, #9271	AB_329825	1:500
Rabbit—Akt	Cell Signalling, #9272	AB_329827	1:1000
Rabbit—P-ERK 1/2 thr-202/tyr-204	Cell Signalling, #9101	AB_331646	1:500
Rabbit—ERK 1/2	Cell Signalling, #9102	AB_330744	1:1000
Rabbit—P-MEK ser-217/221	Cell Signalling, #9121	AB_331648	1:500
Rabbit—MEK	Cell Signalling, #9122	AB_823567	1:1000
Mouse—AP50	BD Biosciences, 611351	AB_398873	1:250
Mouse—Flotillin 1	BD Biosciences, 610820	AB_398139	1:500
Rabbit—Caveolin 1	Cell Signalling, #3238	AB_2072166	1:1000
Rabbit—GAPDH	Cell Signalling, #2118	AB_2072166	1:1000
